# The Peptidoglycan Recognition Protein 1 confers immune evasive properties on pancreatic cancer stem cells

**DOI:** 10.1136/gutjnl-2023-330995

**Published:** 2024-05-16

**Authors:** Juan Carlos López-Gil, Susana García-Silva, Laura Ruiz-Cañas, Diego Navarro, Adrián Palencia-Campos, Antonio Giráldez-Trujillo, Julie Earl, Jorge Dorado, Gonzalo Gómez-López, Ana Monfort-Vengut, Sonia Alcalá, Matthias M Gaida, Sandra García-Mulero, Pablo Cabezas-Sáinz, Sandra Batres-Ramos, Emma Barreto, Patricia Sánchez-Tomero, Mireia Vallespinós, Leah Ambler, Meng-Lay Lin, Alexandra Aicher, Ana García García de Paredes, Carolina de la Pinta, Alfonso Sanjuanbenito, Ignacio Ruz-Caracuel, Mercedes Rodríguez-Garrote, Carmen Guerra, Alfredo Carrato, Guillermo de Cárcer, Laura Sánchez, César Nombela-Arrieta, Elisa Espinet, Víctor Javier Sanchez-Arevalo Lobo, Christopher Heeschen, Bruno Sainz

**Affiliations:** 1 Cancer Stem Cells and Fibroinflammatory Microenvironment Group, Cancer Department, Instituto de Investigaciones Biomédicas (IIBM) Sols-Morreale CSIC-UAM, Madrid, Spain; 2 Biomarkers and Personalized Approach to Cancer Group (BIOPAC), Area 3 Cancer, Instituto Ramón y Cajal de Investigación Sanitaria (IRYCIS), Madrid, Spain; 3 Department of Biochemistry, Autónoma University of Madrid (UAM), Madrid, Spain; 4 Microenvironment and Metastasis Group, Molecular Oncology Programme, Spanish National Cancer Research Centre (CNIO), Madrid, Spain; 5 Biobanco Hospital Ramón y Cajal, Instituto Ramón y Cajal de Investigación Sanitaria (IRYCIS), Madrid, Spain; 6 Grupo de Oncología Cutánea, Servicio de Anatomía Patológica, Hospiral Universitario 12 de Octubre, Instituto de Investigación Sanitaria Hospital 12 de Octubre (imas12), Madrid, Spain; 7 Área Cáncer, Centro de Investigación Biomédica en Red (CIBERONC), ISCIII, Madrid, Spain; 8 Stem Cells and Cancer Group, Clinical Research Programme, Spanish National Cancer Research Centre (CNIO), Madrid, Spain; 9 Bioinformatics Unit, Spanish National Cancer Research Centre (CNIO), Madrid, Spain; 10 Cell Cycle and Cancer Biomarkers Laboratory, Cancer Department, Instituto de Investigaciones Biomédicas (IIBM) Sols-Morreale CSIC-UAM, Madrid, Spain; 11 Institute of Pathology, JGU-Mainz, University Medical Center Mainz, Mainz, Germany; 12 TRON, JGU-Mainz, Translational Oncology at the University Medical Center, Mainz, Germany; 13 Research Center for Immunotherapy, JGU-Mainz, University Medical Center Mainz, Mainz, Germany; 14 Department of Pathology and Experimental Therapy, Universidad de Barcelona Facultad de Medicina y Ciencias de La Salud, Barcelona, Spain; 15 Molecular Mechanisms and Experimental Therapy in Oncology Program (Oncobell), IDIBELL, Barcelona, Spain; 16 Department of Zoology, Genetics and Physical Anthropology, Veterinary Faculty, Universidade de Santiago de Compostela, Lugo, Spain; 17 School of Medicine and Health Sciences, University of Alcalá, Alcalá de Henares, Spain; 18 Barts Cancer Institute, Queen Mary University of London, London, UK; 19 Precision Immunotherapy, Graduate Institute of Biomedical Sciences, China Medical University, Taichung, Taiwan; 20 Gastroenterology and Hepatology, Hospital Universitario Ramon y Cajal, Madrid, Spain; 21 Radiation Oncology, Hospital Universitario Ramón y Cajal, Madrid, Spain; 22 Pancreatic and Biliopancreatic Surgery Unit, Hospital Universitario Ramon y Cajal, Madrid, Spain; 23 Ramon y Cajal University Hospital Anatomy Pathology Service, Madrid, Spain; 24 Molecular Pathology of Cancer Group, Area 3 Cancer, Instituto Ramón y Cajal de Investigación Sanitaria (IRYCIS), Madrid, Spain; 25 Medical Oncology Service, Hospital Universitario Ramón y Cajal, Madrid, Spain; 26 Experimental Oncology Group, Molecular Oncology Programme, Spanish National Cancer Research Centre (CNIO), Madrid, Spain; 27 Department of Medical Oncology and Hematology, University and University Hospital Zurich, Zürich, Switzerland; 28 Grupo de Oncología Molecular, Instituto de Investigaciones Biosanitarias, Facultad de Ciencias Experimentales, Universidad Francisco de Vitoria (UFV), Pozuelo de Alarcón, Spain; 29 Pancreatic Cancer Heterogeneity, Candiolo Cancer Institute – FPO – IRCCS, Candiolo (TO), Italy

**Keywords:** PANCREATIC CANCER, STEM CELLS, IMMUNE RESPONSE, CANCER IMMUNOBIOLOGY

## Abstract

**Objective:**

Pancreatic ductal adenocarcinoma (PDAC) has limited therapeutic options, particularly with immune checkpoint inhibitors. Highly chemoresistant ‘stem-like’ cells, known as cancer stem cells (CSCs), are implicated in PDAC aggressiveness. Thus, comprehending how this subset of cells evades the immune system is crucial for advancing novel therapies.

**Design:**

We used the KPC mouse model (*LSL-Kras^G12D/+^; LSL-Trp53^R172H/+^; Pdx-1-Cre*) and primary tumour cell lines to investigate putative CSC populations. Transcriptomic analyses were conducted to pinpoint new genes involved in immune evasion. Overexpressing and knockout cell lines were established with lentiviral vectors. Subsequent *in vitro* coculture assays, *in vivo* mouse and zebrafish tumorigenesis studies, and *in silico* database approaches were performed.

**Results:**

Using the KPC mouse model, we functionally confirmed a population of cells marked by EpCAM, Sca-1 and CD133 as authentic CSCs and investigated their transcriptional profile. Immune evasion signatures/genes, notably the gene peptidoglycan recognition protein 1 (PGLYRP1), were significantly overexpressed in these CSCs. Modulating PGLYRP1 impacted CSC immune evasion, affecting their resistance to macrophage-mediated and T-cell-mediated killing and their tumourigenesis in immunocompetent mice. Mechanistically, tumour necrosis factor alpha (TNFα)-regulated PGLYRP1 expression interferes with the immune tumour microenvironment (TME) landscape, promoting myeloid cell-derived immunosuppression and activated T-cell death. Importantly, these findings were not only replicated in human models, but clinically, secreted PGLYRP1 levels were significantly elevated in patients with PDAC.

**Conclusions:**

This study establishes PGLYRP1 as a novel CSC-associated marker crucial for immune evasion, particularly against macrophage phagocytosis and T-cell killing, presenting it as a promising target for PDAC immunotherapy.

**WHAT IS ALREADY KNOWN ON THIS TOPIC**

- Although pancreatic ductal adenocarcinoma (PDAC) cancer stem cells (CSCs) have been identified in human tumours, little is known about murine CSCs in PDAC genetically engineered mouse models.
- Cancer cells display immune evasive properties that influence the tumour microenvironment; however, the role of CSCs in immune evasion/suppression is not well understood.

**WHAT THIS STUDY ADDS**

- Murine PDAC CSCs can be identified by combining EpCAM, Sca-1 and CD133 markers, and this population presents enhanced immune evasive properties conferred by peptidoglycan recognition protein 1 (PGLYRP1) expression.
- PGLYRP1 is a novel CSC marker whose overexpression leads to immunosuppression by interfering with TNFα signalling, and its loss renders PDAC cells vulnerable to immune cell-mediated elimination.

**HOW THIS STUDY MIGHT AFFECT RESEARCH, PRACTICE OR POLICY**

- Secreted PGLYRP1 could be used as a PDAC predictive biomarker in patient liquid biopsy samples, as well as a tool to stratify patients at early stages of disease.
- PGLYRP1 emerges as a new target for the development of inhibitors of PDAC, to be used in combination with other approved therapies or immune checkpoint inhibitors.

## Introduction

Pancreatic ductal adenocarcinoma (PDAC) is a highly metastatic and chemo-refractory tumour^1^ with a 5-year survival rate of approximately 11%,^2^ the latter being attributed to a subpopulation of cells within the tumour known as cancer stem cells (CSCs).^3 4^ While CSCs are defined by their exclusive in vivo tumourigenicity, unlimited self-renewal, metastatic capacities and chemoresistance,^5 6^ few studies have addressed their interplay with the immune system. Our group identified that CD47 present on pancreatic CSCs circumvents macrophage (MΦ) phagocytosis^7^ and that metabolically active CSCs modulate known immune checkpoints such as programmed death-ligand 1 (PD-L1),^8^ a characteristic seen in other tumours.^9^ Immune checkpoint blockade has facilitated the development of new treatments that achieve partial or complete responses in cancer patients^10 11^; however, for PDAC, this approach has only produced limited (but promising) results.^12 13^ The latter is likely due to the aforementioned strategies used by CSCs to evade tumour immune surveillance and other immune evasive properties that we are still far from understanding, which likely contribute to the immunologically ‘cold’ phenotype and aggressiveness of PDAC.

In this study, we characterised a population of CSCs in *LSL-Kras^G12D/+^; LSL-Trp53^R172H/+^; Pdx-1-Cre* (KPC) mouse tumours, which exhibited a transcriptomic signature associated with immune evasion. Notably, peptidoglycan recognition protein 1 (*Pglyrp1*) was among the most significantly upregulated genes. Indeed, PGLYRP1 overexpression protected cells from immune-mediated cytotoxic effects, and its knockout (KO) impeded tumour growth in immunocompetent mice. We not only replicated these phenotypes in human models, but we additionally observed a significant increase in PGLYRP1 in patient serum samples, highlighting its possible use as a PDAC biomarker. Taken together, our results shed light on the role of a previously unidentified PDAC CSC-associated marker and immune evasive protein with diagnostic and treatment utility.

## Results

### Isolation of pancreatic cell populations with stemness features

**Figure 1:**
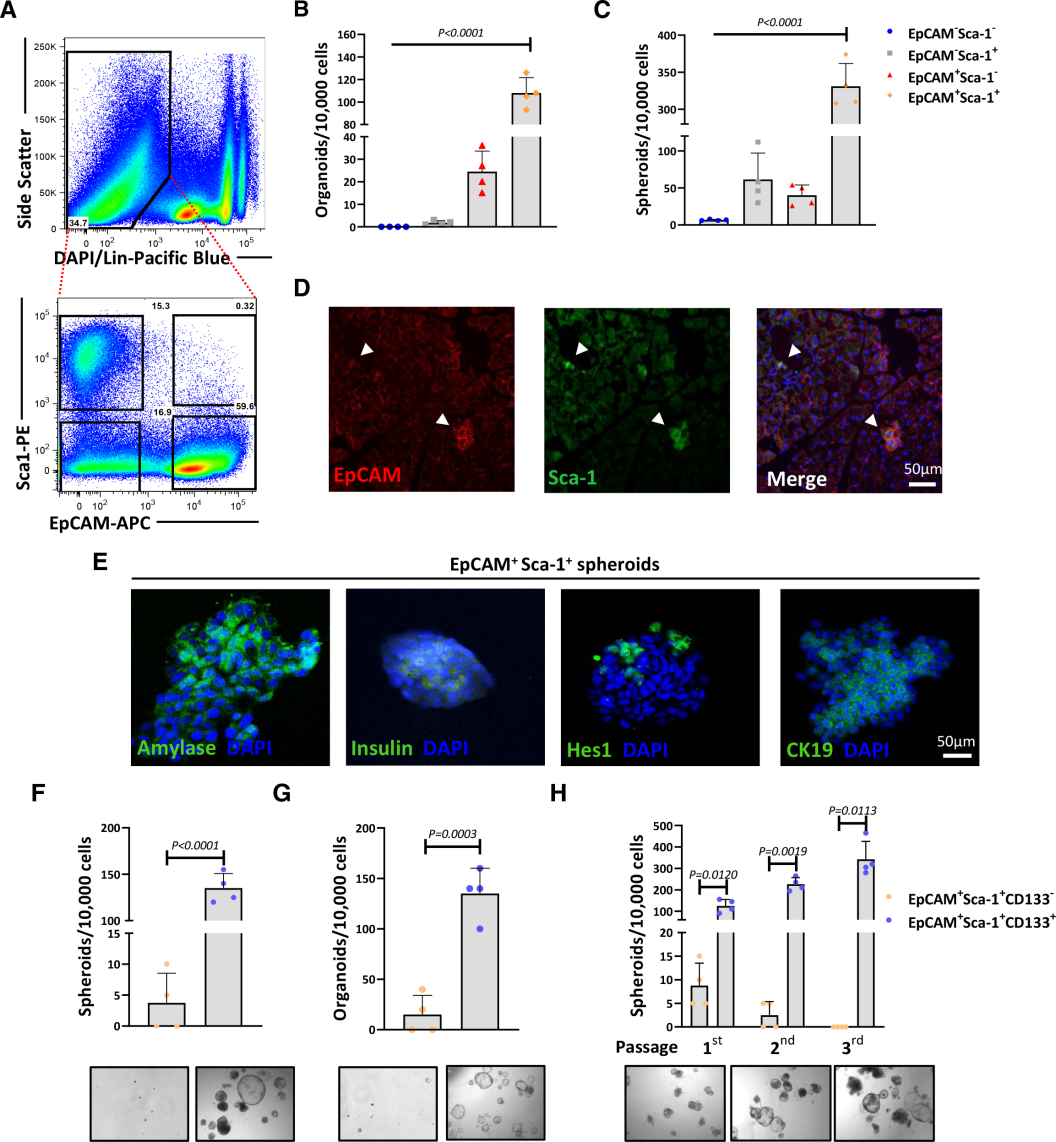
Isolation of pancreatic populations with stemness features. (A) Top panel: representative flow cytometry plot showing the gating strategy for isolation of pancreatic cell populations after lineage depletion from an 8-week-old C57Bl/6J mouse. Cells were stained with anti-CD45 and anti-CD31 (lineage cocktail). Bottom panel: lineage depleted cells were stained with anti-EpCAM and anti-Sca-1 resulting into four populations. (B–C) Quantification of organoid forming capacity in Matrigel (B) or spheroid forming efficiency (C) for the different pancreatic cell subsets identified in (A). Shown are mean organoid numbers/10 000 cells±STDEV or mean spheroid numbers/10 000 cells±STDEV (n=4, p values determined by one-way analysis of variance (ANOVA), with Dunnett’s test). (D) Representative confocal images of normal murine pancreatic tissue showing rare cells positive for EpCAM (red), Sca-1 (green) and DAPI (nuclear marker, blue). Arrows indicate EpCAM^+^Sca-1^+^ populations. Scale=50 µm. (E) Representative confocal images of EpCAM^+^Sca-1^+^ cell-derived spheroids after 10 days of culture. Spheroids were stained with antibodies against pancreatic lineage markers amylase, Hes1, insulin and cytokeratin 19 (CK19) (all in green) and DAPI (nuclear marker, blue). (F–G) Mean spheroid numbers/10 000 cells±STDEV (F) or mean organoids/10 000 cells±STDEV (G) and representative bright field images (bottom) of spheroids or organoids (in Matrigel) generated from sorted EpCAM^+^Sca-1^+^CD133^+^ and EpCAM^+^Sca-1^+^CD133^–^ cells after 10 days of culture (n=4, p values as determined by unpaired t-test). (H) Mean spheroid numbers/10 000 cells±STDEV of EpCAM^+^Sca-1^+^CD133^-^ and EpCAM^+^Sca-1^+^CD133^+^ cell-derived spheroids across serial passages (top) (n=4, p values determined by unpaired t-test). Representative bright field images (bottom) show EpCAM^+^Sca-1^+^CD133^+^ cell-derived spheroids at each passage. DAPI, 4',6-diamidino-2-phenylindole; EpCAM, epithelial adhesion cell adhesion molecule; Sca-1, stem cell antigen 1; STDEV, standard deviation.

To identify cell subpopulations within the mouse pancreas with stem-like potential, various stem cell-related cell surface markers (ie, EpCAM, Sca-1, cMet, CD34, CD49f and CD133) were tested,^14–17^ and only the combination of EpCAM and Sca-1, after stroma depletion (CD45^-^ and CD31^-^ cells), allowed for the separation of four distinct subpopulations (figure 1A, online supplemental figure S1A), which were subjected to spheroid and organoid formation assays. EpCAM^+^Sca-1^+^ cells displayed the highest sphere/organoid formation capacity (figure 1B,C), suggesting stem cell potential. We also detected by immunofluorescence EpCAM^+^Sca-1^+^ cells in normal pancreata sections (figure 1D) and also found that these cells expressed genes related to tissue stemness (online supplemental figure S1B). To test multilineage potential, we analysed lineage markers in spheroids and found amylase (acinar lineage), insulin (islet lineage), Hes1 and cytokeratin 19 (CK19) (both ductal lineage) single-positive cells, indicating that EpCAM^+^Sca-1^+^ cells can give rise to more differentiated cells (figure 1E). We also found that most EpCAM^+^Sca-1^+^ cells were also CD133^+^ (online supplemental figure S1C), and in functional assays, EpCAM^+^Sca-1^+^CD133^+^ cells (ie, triple positive) had the highest spheroid and organoid forming capacity (figure 1F,G). Remarkably, spheroids could be maintained across several generations, indicating extended self-renewal capacity (figure 1H).

10.1136/gutjnl-2023-330995.supp1 Supplementary data

### EpCAM^+^Sca-1^+^ cells expand during carcinogenesis and possess CSC features

**Figure 2:**
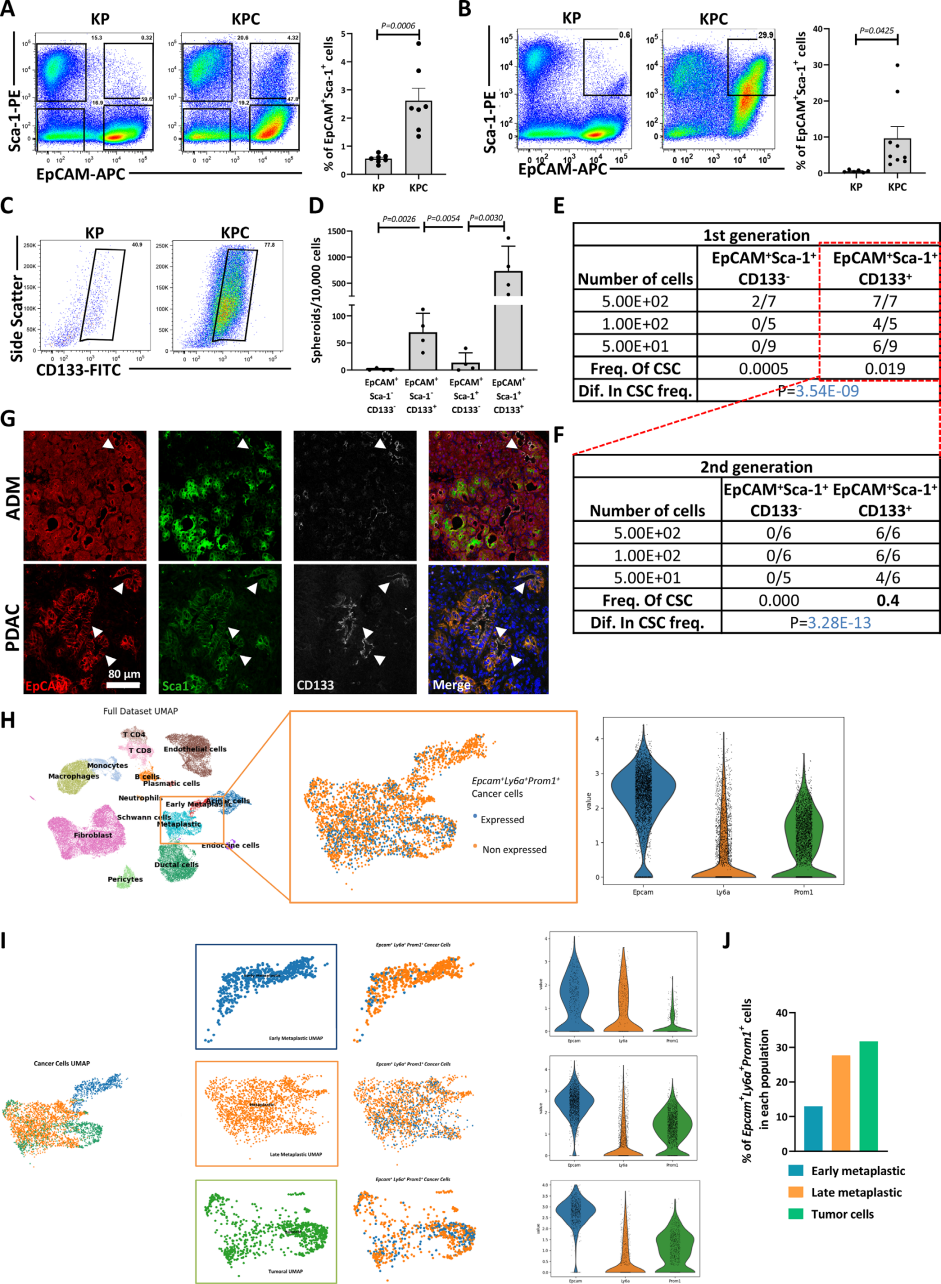
EpCAM^+^Sca-1^+^CD133^+^ cells possess CSC properties and expand during tumour progression. (A) Left panel: representative flow cytometry plot showing Lin^-^ pancreatic cells stained with EpCAM and Sca-1 from 8-week-old control LSL-Kras^G12D/+^; LSL-Trp53^R172H/+^ mice (KP) versus LSL-Kras^G12D/+^; LSL-Trp53^R172H/+^; Pdx-1-Cre mice (KPC). Right panel: histogram plot showing the mean±SEM percentage of EpCAM^+^Sca-1^+^ cells in control KP and KPC animals (n=6, p values determined by unpaired t-test). (B) Left panel: representative flow cytometry plot displaying EpCAM and Sca-1 expression in Lin^-^ cells from a KPC mouse tumour at 16 weeks and corresponding pancreata from a control KP mouse. Right panel: histogram plot showing the mean±SEM percentage of EpCAM^+^Sca-1^+^ cells in KP and KPC groups (n=6, p values determined by unpaired t-test). (C) Representative flow cytometry plot showing the expression of CD133 within the EpCAM^+^Sca-1^+^ gate in a KP and KPC mouse at 16 weeks. (D) Mean±SEM of the number of spheroids/10 000 cells generated by the indicated populations after 10 days in sphere culture conditions. Cells were sorted from the pancreata of 16-week-old KPC mice (n=4, p values determined by one-way analysis of variance (ANOVA), with Dunnett’s test). (E) Panel detailing the tumorigenic potential (number of tumours formed/number of injections) of the indicated number of EpCAM^+^Sca-1^+^ cells injected in the flanks of athymic nude mice. Cells were sorted from KPC tumours and segregated by CD133 expression. Predicted frequency (freq.) of CSCs as a function of the evaluated dilutions are shown (p values determined by χ^2^ analysis obtained using ELDA software). (F) Panel detailing secondary engraftment potential of EpCAM^+^Sca-1^+^CD133^+^ and EpCAM^+^Sca-1^+^CD133^-^ tumour cells isolated from parental tumours generated in (E). Predicted CSC frequencies (freq.) as a function of the evaluated dilutions are shown (no. of injections >5, p values determined by χ^2^ analysis obtained by ELDA software). (G) Representative confocal images showing triple staining with antibodies against EpCAM (red), Sca-1 (green), CD133 (grey) and DAPI (nuclear marker, blue). Top panel: pancreata from a KPC mouse at 8 weeks where acinar-ductal metaplasia (ADM) can be observed. Bottom panel: tumour (PDAC) from a 16-week-old KPC mouse. Arrowheads indicate the triple-positive population. Scale=80 µm. (H) Left panel: UMAP representing the clusters from the lineage tracing single-cell RNA sequencing (scRNA-seq) dataset from Schlesinger *et al*.^21^ Middle panel: the metaplastic cell cluster is amplified with Epcam^+^Ly6a^+^Prom1^+^ cells highlighted in blue, and the percentage of triple-positive cells is indicated. Right panel: expression of the markers in the metaplastic population. (I) Left panels: UMAP corresponding to the different states of early tumorigenesis included in this dataset. Middle panels: amplification of the different states in early tumour evolution. Triple-positive cells are highlighted in blue. Right panels: violin plots showing the levels of expression of the indicated genes in the populations displayed. (J) Histogram representing the percentage of early metaplastic, late metaplastic and tumour cells expressing the three markers from the total in each population. CSC, cancer stem cell; ELDA, extreme limiting dilution assay; EpCAM, epithelial adhesion cell adhesion molecule; Ly6a, lymphocyte antigen 6A; PDAC, pancreatic ductal adenocarcinoma; Prom1, prominin-1; Sca-1, stem cell antigen 1; STDEV, standard deviation; UMAP, Uniform Manifold Approximation and Projection.

In the KPC mouse model of PDAC,^18–20^ all epithelial cell descendants constitutively express active *Kras* and *p53* mutant genes. However, the limited number of malignant lesions suggests that not all cells are equally tumourigenic. We studied if this process would affect the frequency of the above-identified populations. Notably at 8–9 weeks, when acinar-ductal metaplasia (ADM) can be detected, there was a significant increase in EpCAM^+^Sca-1^+^ cells in the pancreata of KPC mice compared with KP controls, while other populations remained mostly unchanged (figure 2A, online supplemental figure S1D, E). At later time points (16 weeks), when PDAC is detectable, expansion of EpCAM^+^Sca-1^+^ cells was more pronounced (figure 2B), and EpCAM^+^Sca-1^+^ cells could be partitioned using CD133 expression, with higher levels observed in KPC vs KP pancreata (figure 2C), translating into higher spheroid forming capacity (figure 2D), higher tumour propagating capacity in an extreme limiting dilution assay (ELDA) (figure 2E) and sustained long-term (ie, serial transplantation) tumorigenicity (figure 2F). Importantly, other cell populations did not give rise to tumours (online supplemental figure S1F), supporting that only triple-positive cells have tumour initiating capacity. Of note, this population could also be detected in ADM and in PDAC lesions from KPC pancreata (figure 2G). Finally, using a lineage tracing single-cell RNA sequencing (scRNA-seq) dataset based on a *Ptf1a*-CreER; LSL-*KRas*-G12D; LSL-*tdTomato* PDAC mouse model,^21^ we found that epithelial cells expressing *Epcam*, *Ly6a*/Sca-1 and *Prom1/*CD133 expand from early metaplastic states to full tumour cells (figure 2H,I), supporting the existence of these cells early on and throughout tumour development. Thus, triple-positive cells possess all the essential characteristics to be considered *bona fide* CSCs.

### Triple-positive CSCs are enriched in stemness and immune evasion signatures

**Figure 3:**
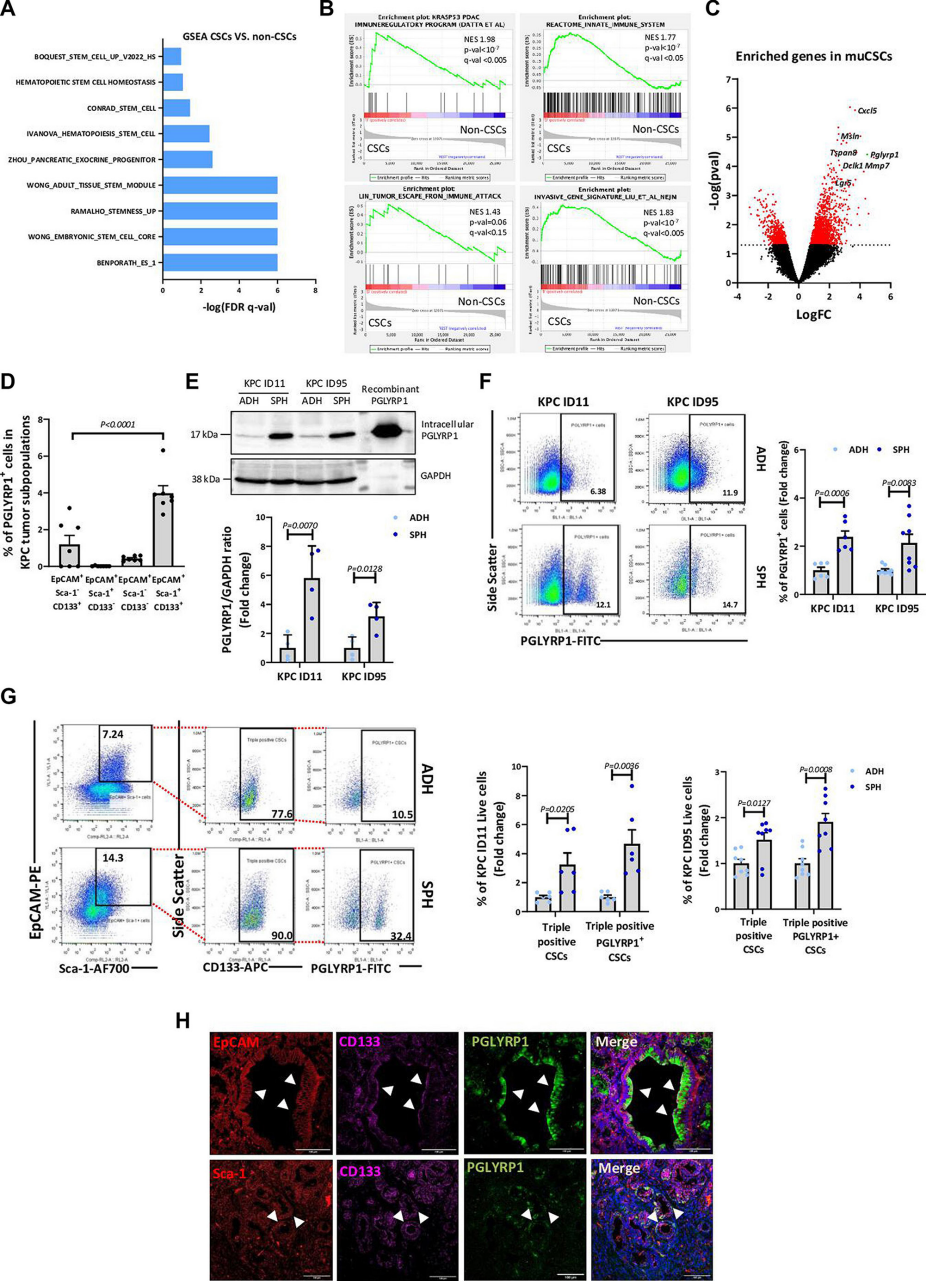
Transcriptomic analysis of triple-positive CSCs and validation of PGLYRP1 as a CSC marker. (A) Gene sets enriched in the transcriptional profile of the EpCAM^+^Sca-1^+^CD133^+^ population (CSCs) versus the rest of populations (non-CSC), showing an enrichment in stem cell signatures. Shown are the -log(FDR q-val) values for each pathway using the indicated published gene signatures, nominal p<0.05, FDR <15% (n=3 biological replicates). (B) GSEA plots showing enrichment of indicated signatures in the EpCAM^+^Sca-1^+^CD133^+^ population (CSC) versus all other tumour cells (non-CSC). (C) Volcano plot showing the significantly enriched genes (in red) in EpCAM^+^Sca-1^+^CD133^+^ CSCs versus all other tumour cells (n=3 biological replicates). (D) Quantification of PGLYRP1^+^ cells in the indicated populations in KPC PDAC tumours detected by flow cytometry, represented as percentage±SEM (n=7, p values determined by one-way ANOVA with Dunnett’s test). (E) Top panel: representative western blot of PGLYRP1 levels from protein extracts derived from adherent (ADH) and spheroid (SPH) cultures. GAPDH was used as an internal loading control. Bottom panel: mean fold change of PGLYRP1/GAPDH densitometric ratios±STDEV, with ADH set as 1.0 (n=3, p values determined by unpaired t-test). (F) Left panel: representative flow cytometry plots showing the percentage of PGLYRP1-FITC expressing cells in both cell lines cultured as ADH and SPH. Right panel: quantification represented as the mean fold change±SEM, with ADH set as 1.0 (n=6, p values determined by unpaired t-test). (G) Left panel: representative flow cytometry plots showing the expansion of the EpCAM^+^Sca-1^+^CD133^+^ CSC and triple-positive PGLYRP1^+^ compartments in KPC cell lines cultured as ADH and SPH. Right panels: frequency of triple-positive and PGLYRP1^+^ triple-positive cells in the indicated cell cultures. Data are represented as the mean fold change in the percentage of the indicated populations±SEM, with ADH set as 1.0 (n=6, p values determined by unpaired t-test). (H) Representative confocal images showing CSC marker expressing cells. EpCAM or Sca-1 (red), CD133 (pink), PGLYRP1 (green) and DAPI (nuclear marker, blue). Arrowheads indicate the CSC population. ANOVA, analysis of variance; CSCs, cancer stem cells; EpCAM, epithelial adhesion cell adhesion molecule; FDR, false discovery rate; FITC, fluorescein isothiocyanate; GAPDH, glyceraldehyde-3-phosphate dehydrogenase; GSEA, gene set enrichment analysis; PDAC, pancreatic ductal adenocarcinoma; PGLYRP1, peptidoglycan recognition protein 1; Sca-1, stem cell antigen 1; STDEV, standard deviation.

Transcriptomic analysis of triple-positive CSCs versus all other tumour cells was performed, and gene set enrichment analysis (GSEA) revealed a significant enrichment in stem cell signatures (figure 3A) and other pathways associated with inflammation and leucocyte migration (online supplemental figure S2A). Importantly, we observed that triple-positive CSCs were also enriched in immune modulation/evasion, innate immune response and tumour invasion signatures (figure 3B). While already described PDAC progression and/or stemness-related genes (ie, *Mmp7*, *Cxcl5, Msln*, *Dckl1*, *Tspan8* and *Lgr5*) were among the most significantly upregulated in CSCs,^22–26^ we also identified, for the first time, *Pglyrp1* as significantly upregulated (figure 3C). In mammals, PGLYRP1 is mainly produced by neutrophils in antibacterial granules, also regulating innate immunity,^27 28^ or by epithelial cells to modulate the microbiome.^29–31^ Recently, PGLYRP1 was associated with immune evasive mechanisms in T cells from certain tumours (ie, melanoma), positioning it as a potential target for cancer treatment.^32^ However, to date, PGLYRP1 has not been studied in PDAC or in PDAC CSCs.

*Pglyrp1* expression was first validated in primary KPC tumours (figure 3D, online supplemental figure S2B), confirming its expression predominantly in the triple-positive population. Next, we cultured primary KPC tumour-derived cell lines in adherence or as spheroids, the latter being a common methodology to enrich for CSCs,^33^ and performed transcriptomic analyses. Again, we observed the previously mentioned signatures and genes (online supplemental figure S2C, D), as well as increased total, secreted and surface-linked PGLYRP1 expression in spheroids from two KPC-derived (ID11 and ID95) cell lines (figure 3E,F, online supplemental figure S2E). Moreover, we found *Pglyrp1* expression in metaplastic cells from the previously mentioned scRNA-seq dataset (online supplemental figure S2F). We also observed enrichment in both triple-positive and PGLYRP1^+^ triple-positive subpopulations in spheroids (figure 3G). In agreement with these data, PGLYRP1 colocalised with EpCAM^+^Sca-1^+^ and EpCAM^+^CD133^+^ cells in KPC tumours (figure 3H).

**Figure 4:**
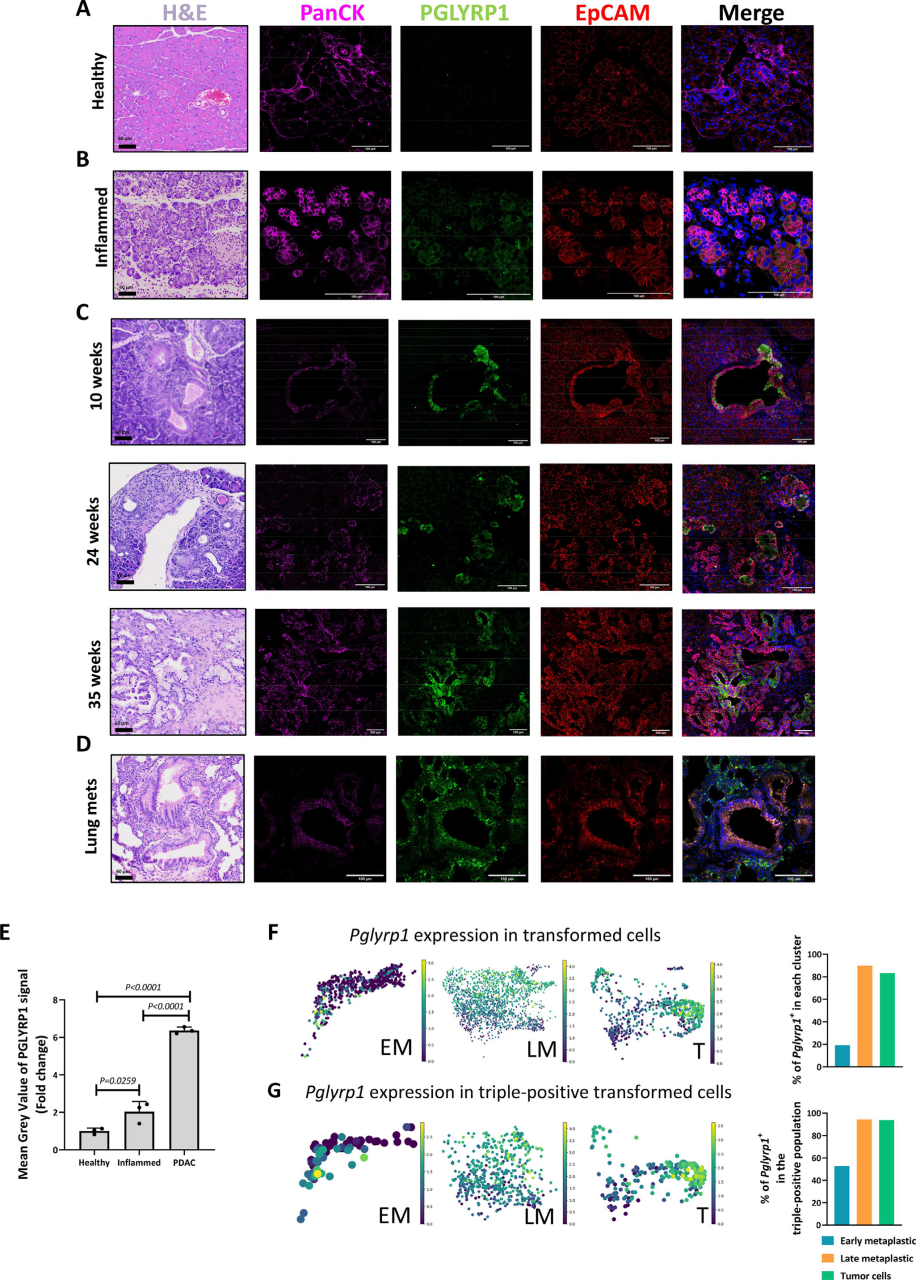
PGLYRP1 expression during tumour evolution. Representative H&E-stained images and confocal microscopy images of PanCK (pink), PGLYRP1 (green), EpCAM (red) and DAPI (nuclear marker, blue) in (A) a mouse healthy pancreata, (B) pancreatic tissue following cerulein-induced pancreatitis, (C) in the pancreas of a 10-week, 24-week and 35-week-old KPC mice and (D) in a lung metastasis (PGLYRP1 colocalises with EpCAM^+^PanCK^+^ lesions; PGLYRP1 staining can also be found in surrounding immune cells). For all images, brightness and contrast were adjusted with ImageJ. Scale bar=60 µm (H&E images) and scale bar=100 µm (fluorescence images). (E) Quantification of PGLYRP1 expression, comparing the mean grey value of the PGLYRP1 staining signal, shown as the mean fold change±STDEV, with healthy pancreas set as 1.0 (n=3, p values determined by one-way ANOVA with post hoc Tukey test). (F) Left panels: UMAPs showing *Pglyrp1* expression in the Schlesinger *et al* dataset^21^ in early metaplastic (EM), late metaplastic (LM) and tumour cell (T) states. Right panel: histogram representing the percentage of *Pglyrp1* expressing cells for each state. (G) Left panels: UMAPs showing *Pglyrp1* expression in the triple-positive population in the Schlesinger *et al* dataset^21^ in early metaplastic (EM), late metaplastic (LM) and tumour cell (T) states. Right panel: histogram representing the percentage of *Pglyrp1* expressing cells in triple-positive cell population for each state. ANOVA, analysis of variance; CSCs, cancer stem cells; DAPI, 4',6-diamidino-2-phenylindole; EpCAM, epithelial adhesion cell adhesion molecule; PanCK, pan-cytokeratin; PGLYRP1, peptidoglycan recognition protein 1; STDEV, standard deviation.

To evaluate the expression of PGLYRP1 during tumour evolution, we combined immunofluorescence analyses with interrogation of single-cell RNA-seq data. In histological sections of the healthy pancreas, we could not detect PGLYRP1 expression in epithelial cells (figure 4A), although in pancreatitis, some acini presented PGLYRP1 (figure 4B). In KPC transformed pancreas; however, PGLYRP1 was detectable as early as 10 weeks and throughout tumour evolution (figure 4C). PGLYRP1 was also expressed in metastatic lung lesions, colocalising with cytokeratins and EpCAM (figure 4D). While PGLYRP1 overexpression is associated with PDAC tumour cells, inflammation can slightly increase its expression in acini (figure 4E). In transcriptomic data, *Pglyrp1* expression increased approximately four times from early metaplastic to late metaplastic cells (figure 4F). Interestingly, nearly half of the triple-positive CSCs expressed *Pglyrp1* in early metaplastic states, increasing to almost 100% in later stages (figure 4G). Taken together, these results suggest that the expression of PGLYRP1 by CSCs and other tumour cells could be relevant for tumour initiation and progression.

### PGLYRP1 is not relevant for stemness but essential for tumour formation

**Figure 5:**
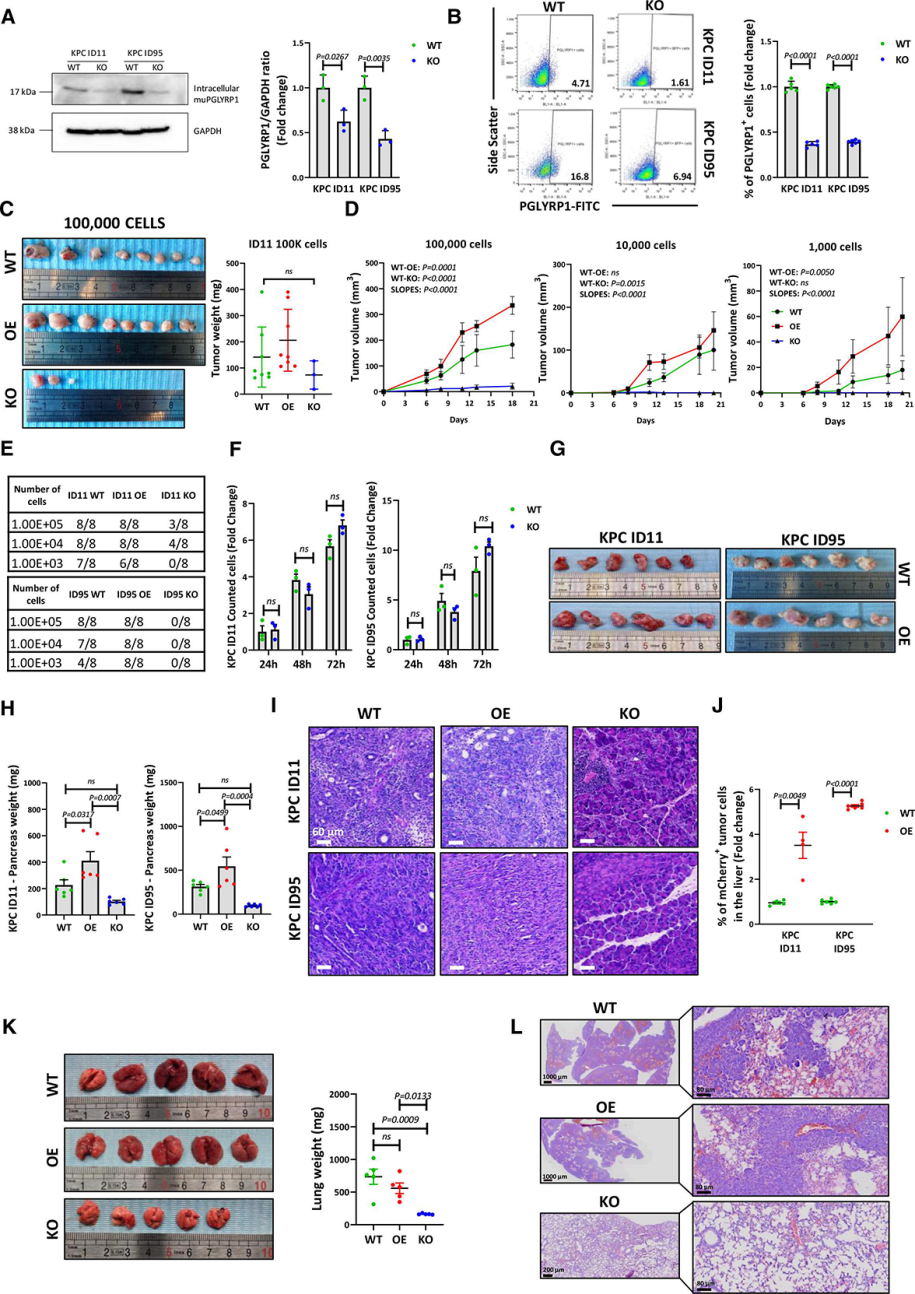
Tumorigenic and metastatic potential of PGLYRP1 knockout and overexpression models. (A) Left panel: representative western blot of intracellular PGLYRP1 levels in protein lysates from wild-type (WT) and CRISPR knockout (KO) KPC ID11 and ID95 cell lines. GAPDH was used as an internal loading control. Right panel: mean fold change of the PGLYRP1/GAPDH densitometric ratios±STDEV, with each WT set as 1.0 (n=3, p values determined by unpaired t-test). (B) Left panel: representative flow cytometry plots of PGLYRP1 expression in both WT and KO cell lines. Right panel: mean fold change in PGLYRP1 expressing cells±SEM, with WT set as 1.0 (n=5, p values determined by unpaired t-test). (C) Left panel: images of tumours obtained from a subcutaneous extreme limiting dilution assay (ELDA). Shown are tumours extracted from immunocompetent C57Bl/6J mice 3 weeks postinjection of 100 000 WT, PGLYRP1 overexpressing (OE) or KO cells derived from the ID11 cell line. Right panel: mean tumour weights (mg)±SEM (no. of injections=8, p values determined by one-way ANOVA with post hoc Tukey test). (D) Growth curves indicating the mean tumour volume (mm^3^)±SEM over 20 days following injection of 100 000, 10 000 or 1000 ID11 wild-type (WT), PGLYRP1 OE or KO cells. The slopes for each group were compared using the ‘comparing slopes tool’ (GraphPad v8), and the p value presented was calculated by comparing all slopes. P values to compare between groups were calculated by two-way ANOVA. (E) Panels detailing the tumorigenic potential of the indicated numbers of injected ID11 and ID95 WT, PGLYRP1 OE or KO cells in immunocompetent C57Bl/6J mice. Each column shows the number of tumours formed/number of injections. (F) Proliferation (no. cells) of KPC ID11 and ID95 WT and PGLYRP1 KO at 24, 48 and 72 hours (h) after seeding, represented as the mean fold change±STDEV with WT 24 hours set as 1.0 (n=3, per condition and time, p values determined by unpaired t-test). (G) Images of tumours at the time of sacrifice from orthotopic injection of 10^4^ ID11 or ID95 WT or OE cells in immunocompetent C57Bl/6J mice. KO cells did not succeed in forming tumours. (H) Mean pancreata weight (mg)±SEM (n=6, p values determined by one-way ANOVA with post hoc Tukey test). (I) Representative H&E-stained sections of orthotopic tumours and pancreata from (G). Scale=60 µm. (J) Mean fold change in the percentage of mCherry^+^ tumour cells in digested livers±STDEV, with WT set as 1.0, as detected by flow cytometry (n=4 for ID11 and n=6 for ID95, p values determined by unpaired t-test). (K) Left panel: images of the lungs at the time of sacrifice from intravenous injection of 10^6^ KPC WT, PGLYRP1 OE or KO cells in immunocompetent C57Bl/6J mice. Right panel: Mean lung weight (mg)±SEM (n=5, p values as determined by one-way ANOVA with post hoc Tukey test). (L) Representative H&E-stained sections of lung metastases from (K). Left images: zoom 10× (scale=1000 µm or 200 µm). Right images: zoom 40× (scale=80 µm). ANOVA, analysis of variance; GAPDH, glyceraldehyde-3-phosphate dehydrogenase; PGLYRP1, peptidoglycan recognition protein 1; STDEV, standard deviation.

To determine if PGLYRP1 is involved in stemness, PGLYRP1 KO cells were generated in KPC ID11 and ID95 cells (figure 5A,B). To maintain the heterogeneity of the primary culture and the CSC compartment, CRISPR KO validation was performed on the cell pool instead of from a single-cell-derived culture. A consistent reduction in PGLYRP1 expression was confirmed. We next performed an ELDA of wild-type (WT) and KO cells in C57Bl/6J immunocompetent mice. In addition, PGLYRP1 overexpressing (OE) cells were generated (online supplemental figure S3A) and included. While no relevant differences in tumour numbers or weights between WT and OE cells were observed (figure 5C), OE cell-derived tumours grew significantly faster (figure 5D, online supplemental figure S3B, C), and many presented a less differentiated appearance in histological analyses (online supplemental figure S3D). Strikingly, PGLYRP1 KO significantly impaired tumour take (figure 5E). For ID11, no tumours formed when 10^3^ KO cells were injected, and for ID95, KO cells were unable to form tumours at all dilutions (figure 5D,E, online supplemental figure S3B–D). To discard differences in PGLYRP1 KO cell proliferation, we quantified cell numbers in vitro over 3 days and observed no differences compared with WT cells (figure 5F). Importantly, using ID95 cells expressing Cas9 and a control Scramble guide, we could discard that the effects observed for PGLYRP1 KO cells in vivo were due to a CRISPR/Cas9 immunogenic effect as no differences between WT and Scramble cells were observed (online supplemental figure S3E).

To investigate the potential influence of the immune system and further study the possible role of PGLYRP1 in PDAC stemness, we performed an additional tumour formation assay in immunodeficient NOD.CB17-Prkdc^scid/scid^/Rj (NOD.SCID) mice injecting 10^3^ cells, the limiting dilution obtained above. In this setting, KO cells generated the same number of tumours compared with WT and OE cells (online supplemental figure S3F). Although ID11 KO cells grew significantly slower than WT cells, tumours did not show significant diferences in final average weight and volume, which was replicated with the ID95-derived cell lines, suggesting that PGLYRP1 does not impair tumour initiation/stemness and that adaptive immune cells and fully functional innate immune cells were likely responsible for impeding KO cell growth in the immunocompetent setting (online supplemental figure S3G). Moreover, these data discarded a possible role for PGLYRP1 in stemness, although they suggest that PGLYRP1 is an important putative immune evasion protein used by CSCs. Following this idea, we investigated if PGLYRP1 expression favoured primary tumour formation and metastatic colonisation after orthotopic implantations in C57Bl/6J mice pancreata. PGLYRP1 overexpression increased primary tumour size (figure 5G,H) and induced a more dedifferentiated phenotype as determined by histological analysis (figure 5I). Moreover, disseminated cells from OE tumours colonised the liver more efficiently (figure 5J). Again, KO cells did not form tumours or metastases (figure 5G–J). To bypass tumour formation as a requisite for distant organ colonisation, 10^6^ WT, OE and KO cells were intravenously injected in C57Bl/6J mice. PGLYRP1 WT and OE cells succeeded in forming metastases in the lungs, while KO cells did not (figure 5K,L). Altogether, these data support the hypothesis that PGLYRP1 is necessary for initiation and metastasis in immunocompetent mice.

### PGLYRP1 confers immune protection and alters the immune tumour microenvironment

**Figure 6:**
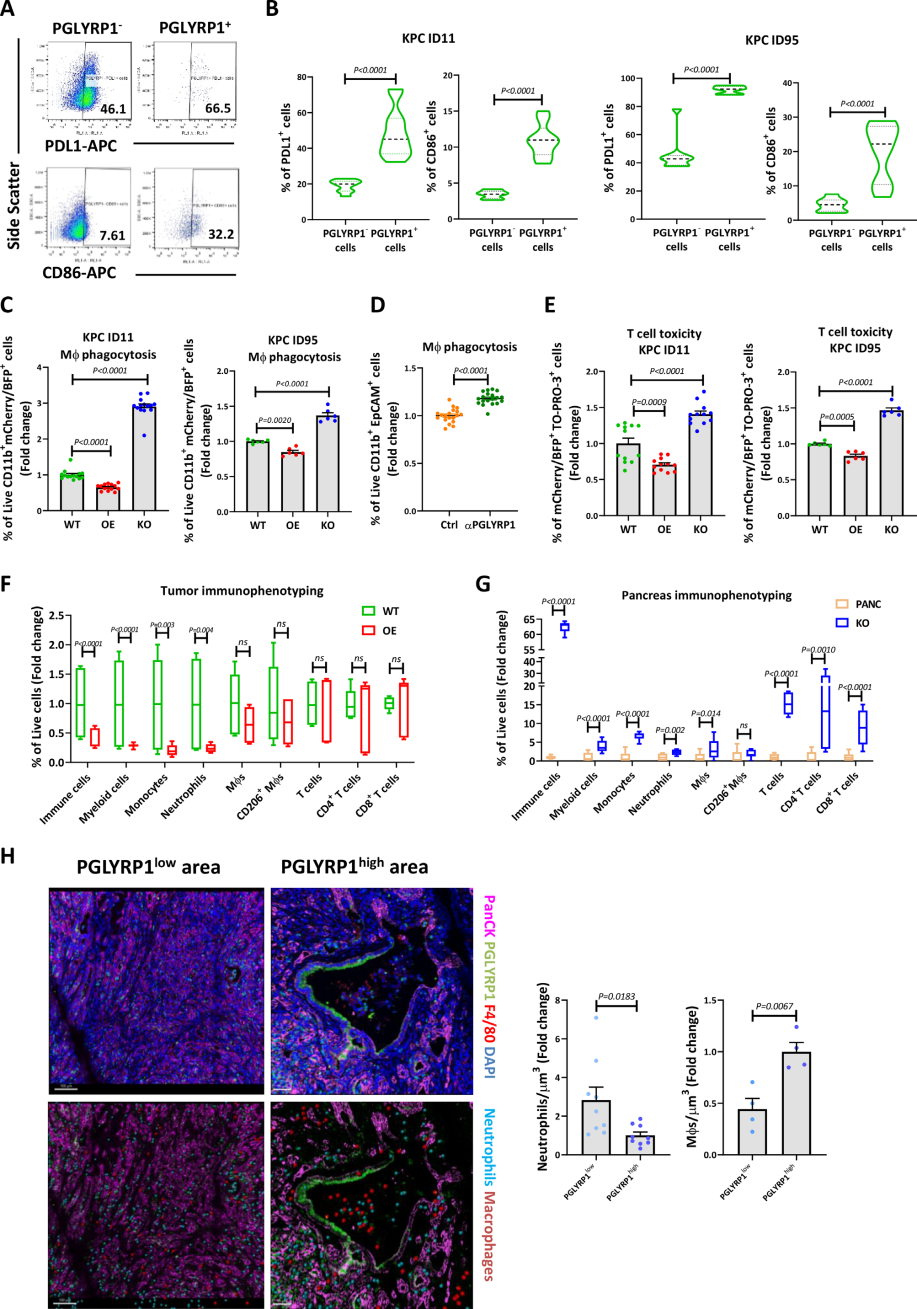
PGLYRP1 promotes immune evasion and alters immune infiltration. (A) Representative flow cytometry plots of PD-L1 and CD86 expression in PGLYRP1^-^ or PGLYRP1^+^ populations. (B) Violin plots representing the quantification of the mean percentage of PD-L1^+^ and CD86^+^ cells in the PGLYRP1^-^ or PGLYRP1^+^ populations for the ID11 cell line (left) and the ID95 cell line (right) determined by flow cytometry (n=9, p values determined by unpaired t-test). (C) Quantification of MΦ-phagocytosed cells (ID11, left; ID95, right), determined by flow cytometry as double-positive (mCherry/BFP^+^ and CD11b^+^) live cells, represented as mean fold change±SEM, with WT set as 1.0 (n=14 for ID11 and n=6 for ID95, p values determined by one-way ANOVA with post hoc Tukey test). Bone marrow-derived primary MΦs were obtained from three different donor mice. (D) Quantification of MΦ-phagocytosed PGLYRP1 WT cells (Ctrl) without or with anti-PGLYRP1 (αPGLYRP1) treatment (0.5 µg/mL; 24 hours), determined by flow cytometry as double-positive (EpCAM^+^ and CD11b^+^) live cells, represented as mean fold change±SEM, with Ctrl set as 1.0 (n=19, p values determined by unpaired t-test). (E) Quantification of dead cells (TO-PRO-3^+^) in the tumour population (mCherry^+^/BFP^+^) for ID11 (left) and ID95 (right) cells, determined by flow cytometry and represented as mean fold change±SEM, with WT set as 1.0 (n=12 for ID11 and n=6 for ID95, p values determined by one-way ANOVA with post hoc Tukey test). T cells were obtained from lymph nodes and spleen of three different donor mice. (F) Percentage of the different indicated immune cell populations in the TME from WT or OE tumours, determined by flow cytometry and represented as box plots (n=3 mice per condition and cell line, p values determined by unpaired t-test). (G) Percentage of the different indicated immune cell populations in the pancreata microenvironment from control or KO-cell-injected pancreata, determined by flow cytometry and represented as box plots (n=3 mice per condition, p values as determined by unpaired t-test). (H) Left panel: representative images of PGLYRP1^low^ and PGLYRP1^high^ tumour areas from spontaneous KPC tumours obtained by 3D quantitative confocal microscopy. Top images show a conventional IF staining, while bottom images show a postprocessed image where neutrophils (light blue) and macrophages (burgundy) are annotated through Imaris spots function for quantification. Right panels: number of indicated immune cells±SEM (neutrophils or macrophages) per µm^3^ in both tumour areas based on PGLYRP1 expression (low or high) (n=9 tumours for neutrophils and n=3 tumours for macrophages, p values determined by unpaired t-test). ANOVA, analysis of variance; EpCAM, epithelial adhesion cell adhesion molecule; IF, immunofluorescence; KO, knockout; MΦ, macrophage; OE, overexpressing; PD-L1, programmed cell death ligand 1; PGLYRP1, peptidoglycan recognition protein 1; TME, tumour microenvironment; WT, wild type.

In line with an immune evasive role, we found that PGLYRP1^+^ cells also coexpressed higher levels of PD-L1 and CD86 compared with PGLYRP1^-^ cells (figure 6A,B), both of them immune checkpoint ligands that engage with PD-1 and cytotoxic T-lymphocyte-associated antigen 4 (CTLA-4) on T cells, respectively, and inhibit T cell activation and antitumour responses,^34^ indicating an overlap between PGLYRP1 and established immune checkpoint ligands. To directly test the immunoevasive capacity, WT, OE and KO cells were cocultured with MΦs and activated T cells to evaluate phagocytosis and cytotoxicity, respectively. We found that KO cells were phagocytosed more efficiently and were more vulnerable to cytotoxic killing than WT cells, while OE cells were more resistant to immune cell attack (figure 6C–E). Supporting these results, anti-PGLYRP1 antibodies increased KPC WT susceptibility to MΦ-mediated phagocytosis (figure 6D). Thus, PGLYRP1 expression seems to have an active role in immune evasion in vitro and may condition the immune landscape in vivo. To test the latter, we performed immune phenotyping in the orthotopic tumours established above and observed a significant decline in immune cell infiltration, particularly in the myeloid cell, monocyte and neutrophil subsets in OE versus WT tumours (figure 6F). However, there were no significant changes in MΦs, CD206^+^ MΦs, general T cells, and CD4^+^ and CD8^+^ T-cell subsets in OE tumours (figure 6F), indicating that PGLYRP1 does not likely influence the infiltration or retention of these immune subsets. In PGLYRP1 KO cell-injected pancreata (in which tumours did not form), we could only evaluate the immune cells that remained after tumour cell clearance, which we compared against the pancreata of control mice. All the immune populations analysed, except for CD206^+^ TAMs, augmented in comparison with healthy controls, indicating the presence of a robust immune infiltration/response against injected KO cells (figure 6G). Thus, PGLYRP1 expression seems to modulate the immune response during tumour formation by altering immune cell infiltration and conferring resistance to activated T cells and MΦs.

To assess the expression and impact of PGLYRP1 on the PDAC immune compartment, the stroma cell populations expressing PGLYRP1 were analysed using the previously mentioned scRNA-seq dataset.^21^ Neutrophils, monocytes, CD4^+^ and CD8^+^ T cells were found to express *Pglyrp1*, although in a lower proportion compared with tumour cells (online supplemental figure S4A, B). Histological sections of KPC tumours confirmed neutrophils as the primary immune cell expressing PGLYRP1 in PDAC (online supplemental figure S4C). Although there is a reduction in neutrophil numbers in OE tumours, a transcriptional analysis of these tumour-associated neutrophils (TANs) revealed that OE-infiltrated TANs express lower levels of migration-related genes (eg, *Cxcr2* and *Cxcr4*) and, interestingly, higher levels of immunosuppressive molecules, including *Pglyrp1* (online supplemental figure S5A), compared with WT tumours. Reduced neutrophil motility in a transwell migration assay (online supplemental figure S5B) and decreased surface expression of CXCR2 and CD95 by flow cytometry in recombinant (r)PGLYRP1-stimulated neutrophils supported these findings (online supplemental figure S5C). Additionally, PGLYRP1 alone enhanced neutrophil survival similar to lipopolysaccharide (LPS), and conditioned medium (CM) from KPC OE cells increased neutrophil survival compared with KPC WT CM (online supplemental figure S5D). M0 MΦs exposed to rPGLYRP1 did not polarise to an M1 or M2 phenotype (online supplemental figure S5E), but when M1-polarised or M2-polarised MΦs were treated with rPGLYRP1, *Cd86* expression was reduced in all MΦ subtypes and *Arg1* expression increased in M2 MΦs (online supplemental figure S5F). In MΦs, CD86 serves as a costimulatory molecule that activates T cells via the interaction with CD28. Consequently, rPGLYRP1 may diminish the immunogenicity of these cells by downregulating CD86 expression. However, the presence of CTLA-4 on T cells alters the function of CD86 towards inducing T cell anergy. Despite its reduced expression, CD86 remains active in MΦs. Moreover, PGLYRP1 exposure also induced the expression of *Camp*, which has been shown to activate CSCs,^35^ reduced the expression of *Tnf* (online supplemental figure S5G) and increased MΦ migration (online supplemental figure S5H), despite no significant effects on MΦ infiltration in PGLYRP1 OE tumours in vivo. In light of the aforementioned results, we used 3D quantitative confocal microscopy to further analyse the spatial distribution of neutrophils and MΦs in KPC spontaneous tumours. Areas with high PGLYRP1 expression exhibited reduced neutrophil but higher MΦ infiltration (figure 6H). These findings support the in vitro migration results and open up the question regarding the role of MΦs in PGLYRP1 expression, given their increased presence in PGLYRP1^high^ areas.

Regarding T cells, exposure of activated and non-activated T cells to rPGLYRP1 showed no variation in exhaustion markers (online supplemental figure S5I). However, activated T cells exhibited reduced viability compared with non-activated T cells (online supplemental figure S5J), highlighting a possible mechanism by which PGLYRP1 protects cells from cytotoxic T-cell mediated death. Overall, these data support the hypothesis of an immunomodulating role for PGLYRP1 promoting an immunosuppressive environment through reduced migration of neutrophils, increased presence of protumoral macrophages and possibly direct induction of activated T cell death.

### TNFα induces PGLYRP1 expression, protecting CSCs from immune clearance

**Figure 7:**
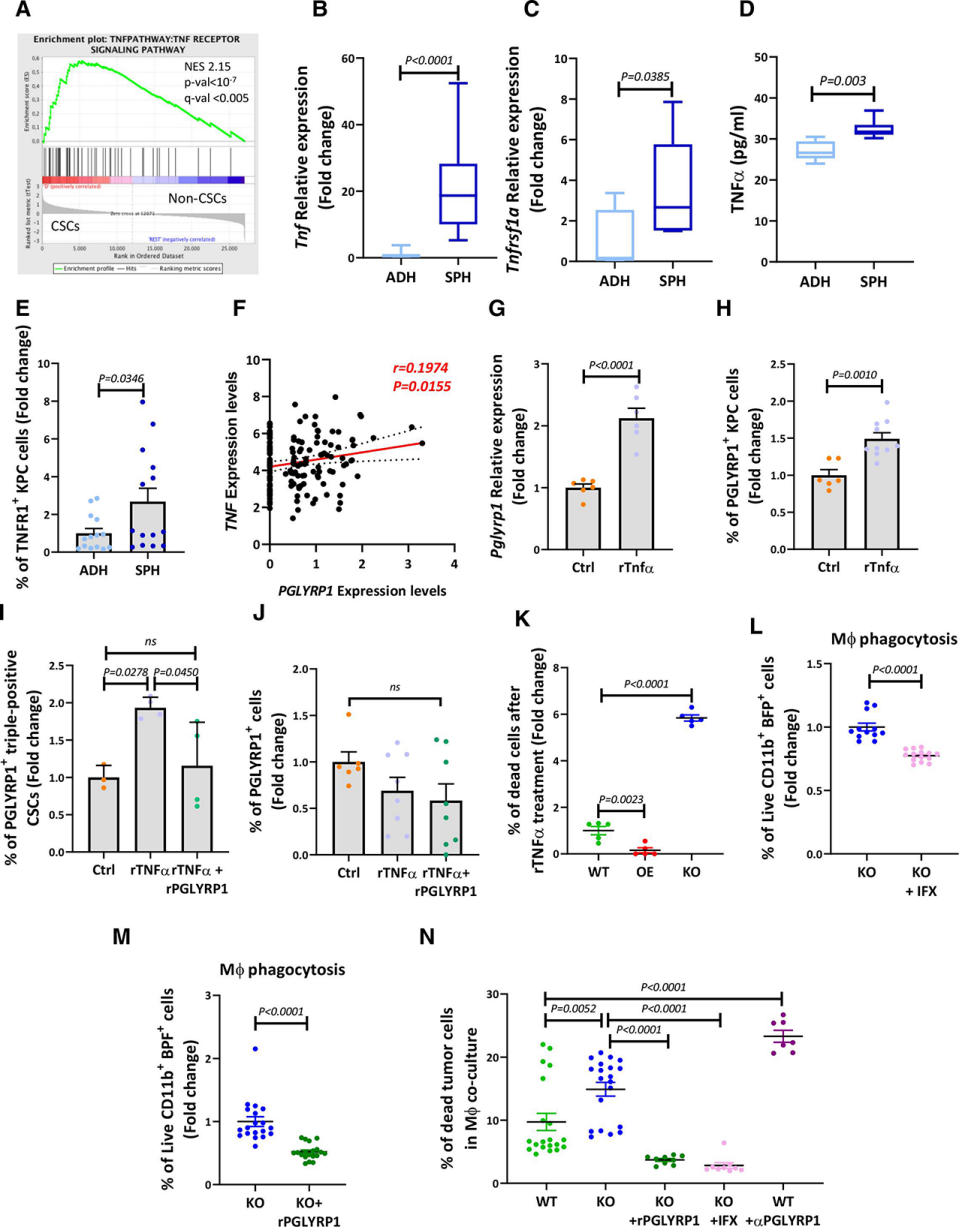
TNFα induces PGLYRP1 expression, protecting CSCs from immune clearance. (A) GSEA plot representing enrichment of the TNF-TNFR signalling pathway from transcriptomic data generated from tumour-isolated triple-positive CSCs versus non-CSCs. (B) RT-qPCR analysis of *Tnf* mRNA expression in KPC cells in adherent monolayer (ADH) or spheroid (SPH) culture conditions. Shown is the mean fold change±SEM, with ADH set as 1.0 (n=4 for ADH and n=5 for SPH, p values determined by unpaired t-test). (C) RT-qPCR analysis of *Tnfrsf1a* mRNA expression in KPC ADH or SPH cultures. Shown is the mean fold change±SEM, with ADH set as 1.0 (n=3, p values determined by unpaired t-test). (D) Box plots representing the mean levels of soluble TNFα (pg/ml) as determined by ELISA comparing KPC ADH versus SPH cell conditioned medium (n=6, p values determined by unpaired t-test). (E) Quantification of flow cytometric analyses of TNFR1^+^ KPC cells grown in ADH or SPH conditions. Shown is the mean fold change±SEM, with ADH set as 1.0 (n=7, p values determined by unpaired t-test). (F) Correlation dot plot of *TNF* and *PGLYRP1* expression in the TCGA database. P value and r were calculated employing Pearson’s correlation. (G) RT-qPCR analysis of *Pglyrp1* mRNA expression in KPC cells after recombinant (r)TNFα stimulation (20 ng/mL) for 6 hours. Shown is the mean fold change±SEM, with Ctrl set as 1.0 (n=6 for controls and n=12 for TNFα treated, p values determined by unpaired t-test). (H) Quantification by flow cytometry of PGLYRP1^+^ cells after treatment with rTNFα (20 ng/mL, 6 hours). Shown is the mean fold change±SEM, with Ctrl set as 1.0 (n=6 for control and n=12 for rTNFα treated, p values determined by unpaired t-test). (I) Quantification by flow cytometry of PGLYRP1^+^ triple-positive CSCs after treatment with rTNFα (20 ng/mL; 6 hours) and rTNFα + rPGLYRP1 (20 ng/mL+1 µg/mL; 6 hours). Shown is the mean fold change±STDEV, with Ctrl set as 1.0 (n=3 for controls and n=4 for treated group, p values determined by one-way ANOVA with post hoc Tukey test). (J) Quantification by flow cytometry of PGLYRP1^+^ non-CSCs after treatment with rTNFα (20 ng/mL; 6 hours) and rTNFα + rPGLYRP1 (20 ng/mL+1 µg/mL; 6 hours). Shown is the mean fold change±STDEV, with Ctrl set as 1.0 (n=3 for controls and n=4 for treated group, p values determined by one-way ANOVA with Dunnett’s test). (K) Quantification of dead cells (TO-PRO-3^+^) after 6 hours of TNFα (20 ng/mL) treatment of KPC WT, PGLYRP1 OE or KO cultures, determined by flow cytometry. Data are represented as the mean fold change±SEM, with WT set as 1.0 (n=5, p values determined by one-way ANOVA). (L) Quantification of MΦ-phagocytosed PGLYRP1 KO cells without or with infliximab (IFX) treatment (10 µg/mL; 24 hours), determined by flow cytometry as double-positive (BFP^+^ and CD11b^+^) live cells, represented as mean fold change±SEM, with KO set as 1.0 (n=12 for KO and n=14 for KO+IFX, p values determined by unpaired t-test). (M) Quantification of MΦ-phagocytosed PGLYRP1 KO cells without or with rPGLYRP1 treatment (1 µg/mL; 24 hours), determined by flow cytometry as double-positive (BFP^+^ and CD11b^+^) live cells, represented as mean fold change±SEM, with KO set as 1.0 (n=19, p values determined by unpaired t-test). (N) Quantification of MΦ-induced cell death in WT and PGLYRP1 KO cells, determined by flow cytometry as EpCAM^+^ or BFP^+^ dead cells, in basal conditions or KO cells treated with rPGLYRP1, infliximab (IFX) or WT cells treated with anti-PGLYRP1 antibody (αPGLYRP1) represented as percentage±SEM (n=20 for WT and KO, 9 for KO+rPGLYRP1 and IFX, and 7 for WT+ αPGLYRP1, p values determined by unpaired t-test). ANOVA, analysis of variance; CSCs, cancer stem cells; GSEA, gene set enrichment analysis; KO, knockout; MΦ, macrophage; rPGLYRP1, recombinant peptidoglycan recognition protein 1; RT-qPCR, reverse-transcription quantitative PCR; STDEV, standard deviation; TCGA, the cancer genome atlas; TNFα, tumour necrosis factor alpha; TNFR, tumour necrosis factor receptor; WT, wild type.

Our RNA microarray data highlighted an enrichment in the TNFα-TNFR1 signalling pathway in triple-positive CSCs (figure 7A and online supplemental figure S6A), including *Tnf* and *Tnfrsf1a* upregulation, which we validated at the mRNA, protein and/or cell surface levels using CSC-enriched spheres or PGLYRP1^+^ triple-positive cells (figure 7B–E, online supplemental figure S6B). Interestingly, a functional connection between TNFα and PGLYRP1 has been described recently.^36^ Bushal *et al* have shown that in microglia, TNFα induces the expression of PGLYRP1. In agreement, another study using oesophageal cancer cells showed that PGLYRP1 modulates TNFα-tumour necrosis factor receptor 1 (TNFR1) signalling by binding TNFR1.^37^ Thus, we hypothesised that TNFα might regulate PGLYRP1 in PDAC CSCs. Indeed, The Cancer Genome Atlas (TCGA) data^38^ showed a positive correlation between *TNF* and *PGLYRP1* expression, supporting a putative functional connection (figure 7F). To experimentally dissect this plausible link, we exposed KPC cells to recombinant (r)TNFα, resulting in increased *Pglypr1* mRNA expression (figure 7G) and frequency of PGLYRP1^+^ cells (figure 7H) along with an expansion in PGLYRP1^+^ CSCs, which was counteracted by the addition of rPGLYRP1 (figure 7I), likely via its binding to TNFR1. Importantly, the non-CSC population did not increase PGLYRP1 expression on rTNFα stimulation (figure 7J). The observed competition with TNFα was confirmed by assessing the expression of TNFα-induced genes in KPC cells treated with rPGLYRP1 (online supplemental figure S6C) and was further validated by using infliximab (TNFα inhibitor) to reduce basal PGLYRP1 expression in spheroid conditions (online supplemental figure S6D). Thus, the sum of these data links PGLYRP1 induction in PDAC CSCs with increased TNFα-TNFR1 signalling.

We next explored TNFα as a potential cytotoxic mediator shaping tumour evolution and the role of PGLYRP1 in regulating/counteracting this effect. Exposure of WT, PGLYRP1 OE and KO cells to rTNFα decreased KO cell viability compared with WT cells, but PGLYRP1 OE cells were significantly protected (figure 7K). While WT cells were also susceptible to the cytotoxic effect of rTNFα, the addition of rPGLYRP1 counteracted this phenotype (online supplemental figure S6E). These results suggested that PGLYRP1 is not only induced by TNFα, but it also serves as a protective mechanism against TNFα-derived cytotoxic effects, promoting tumour cell survival. If this hypothesis is true, the blockade of TNFR1 signalling with the TNFα inhibitor infliximab or the presence of rPGLYRP1 should have an impact on MΦ-mediated phagocytosis of PGLYRP1 KO cells. Indeed, both approaches reduced phagocytosis (figure 7L,M). These results were further supported by the reduced viability of PGLYRP1 KO KPC cells when treated with MΦ CM (online supplemental figure S6F), which contains MΦ-secreted TNFα.^39^ Indeed, when KPC PGLYRP1 KO cells were cocultured with MΦs in the presence of rPGLYRP1 or infliximab, reduced cell death was observed, while when KPC WT cells were cocultured with MΦs in the presence of anti-PGLYRP1 antibodies, increased cell death was observed (figure 7N). In summary, PDAC cells, particularly CSCs, upregulate PGLYRP1 in response to inflammatory signals, notably TNFα from both tumour and tumour microenvironment (TME) cells (eg, MΦ), as a strategy to mitigate its cytotoxic effects by blocking TNFα/TNFR1 signalling.

### PGLYRP1 is present in human PDAC cells, enhances immune evasion and is a potential PDAC biomarker

**Figure 8:**
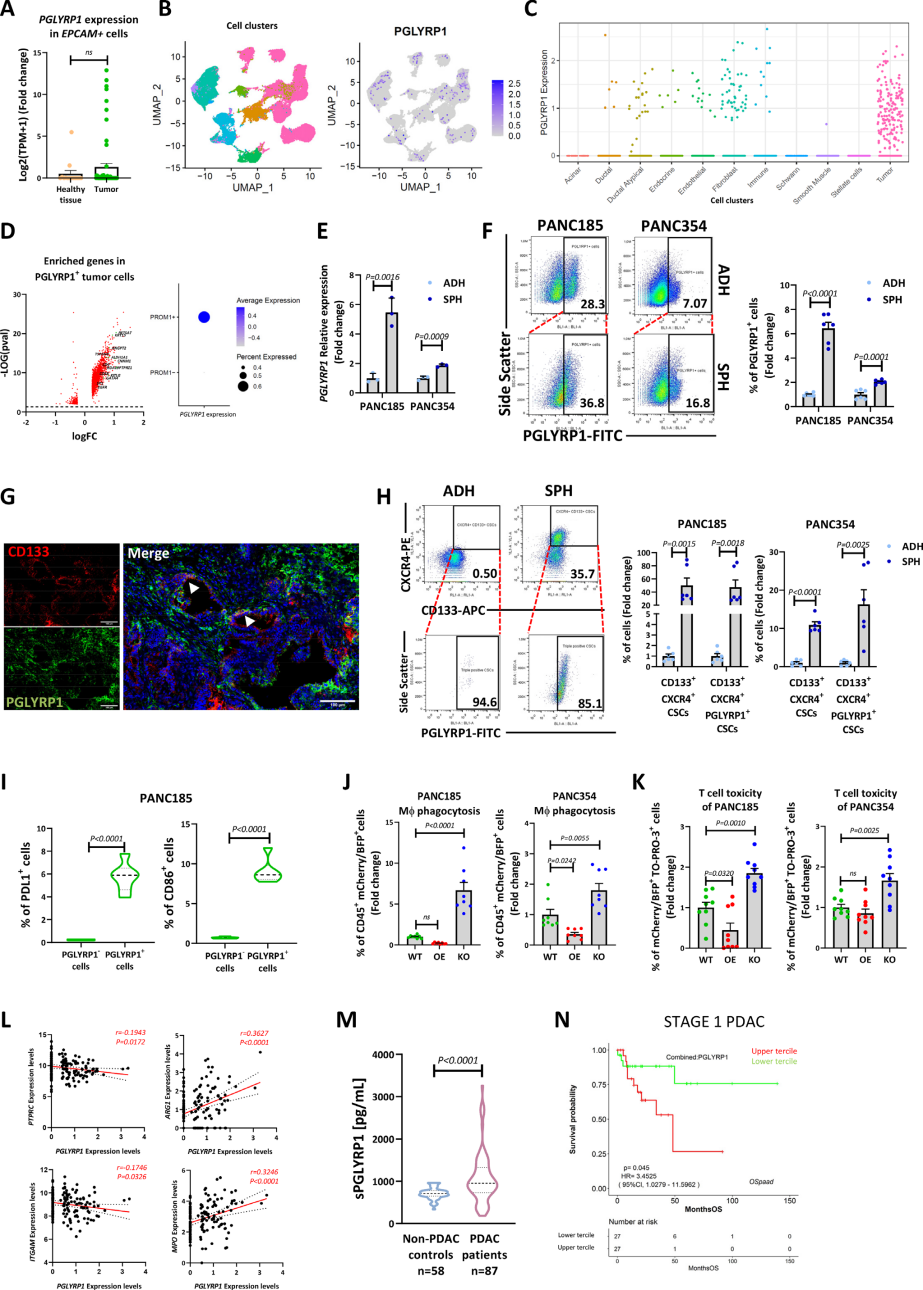
PGLYRP1 in human PDAC CSCs. (A) Mean fold change±SEM of *PGLYRP1* expression in freshly sorted EPCAM^+^ cells from human tumours (tumour) or healthy adjacent pancreatic tissue (healthy tissue), determined by RNA-seq (Espinet *et al* dataset^40^) and with healthy tissue set as 1.0 (n=14 for healthy tissue and n=62 for tumours, p values determined by unpaired t-test). (B) Left panel: UMAP of the different cell clusters present in the Hwang *et al* scRNA-seq dataset of human PDAC.^41^ Right panel: UMAP of *PGLYRP1* expressing cells in the different clusters. (C) Representation of *PGLYRP1* expressing events in each cluster. (D) Left panel: volcano plot showing the significantly enriched genes (in red) in tumour *PGLYRP1* expressing versus *PGLYRP1* non-expressing cells. Genes related to stemness, tumour aggressiveness and chemoresistance are labelled and coloured in green (significant genes, FC >0.25 and p<0.05). Right panel: *PGLYRP1* expression in tumour cells according to *PROM1* expression. (E) Mean fold change±STDEV of *PGLYRP1* mRNA expression in two PDX-derived primary human PDAC cell lines (PANC185 and PANC354) cultured as adherent monolayers (ADH) or spheroids (SPH), determined by RT-qPCR and with ADH set as 1.0 (n=3, p values determined by unpaired t-test). (F) Left panel: representative flow cytometry plots of the percentage of PGLYRP1^+^ cells in ADH or SPH cultures. Right panel: quantification of the mean fold change±SEM of PGLYRP1^+^ cells in ADH or SPH cultures, with ADH set as 1.0 (n=3, p values as determined by unpaired t-test). (G) Representative confocal images of human PDAC tumour stained with CD133 (red), PGLYRP1 (green) and DAPI (blue). Arrowheads indicate double-positive cells. Scale=100 µm. (H) Left panel: representative flow cytometry plots of the percent of CXCR4^+^CD133^+^ CSCs (top) or CXCR4^+^CD133^+^PGLYRP1^+^ (bottom) cells in human PDAC cells grown as ADH or SPH cultures. Right panels: quantification of the indicated populations in PANC185 and PANC354 cultures. Data shown as the mean fold change±SEM, with ADH set as 1.0 (n=3, p values determined by unpaired t-test). (I) Violin plots representing the mean percentage of PDL1^+^ or CD86^+^ cells in the PGLYRP1^-^ or PGLYRP1^+^ populations in PANC185 cells (n=6 for all groups, p values determined by unpaired t-test). (J) Mean fold change±SEM of MΦ-phagocytosed cells determined by flow cytometry as double-positive (mCherry^+^/BFP^+^ and CD45^+^) live events for both PANC185 and PANC354, with WT set as 1.0 (n=8, p values determined by one-way ANOVA). Primary MΦs were obtained from five different healthy donors. (K) Mean fold change±SEM of dead cells (TO-PRO-3^+^) in the tumour population (mCherry^+^/BFP^+^) for both PANC185 and PANC354, determined by flow cytometry and represented with WT set as 1.0 (n=9, p values determined by one-way ANOVA with post hoc Tukey test). T cells were obtained from five different healthy donors. (L) Correlation dot plot of *PTPRC* (CD45), *ITGAM* (CD11b), *MPO*, *ARG1* and *PGLYRP1* expression in the TCGA database. P value and R were calculated employing Pearson’s correlation. (M) Violin plots representing the mean levels of soluble PGLYRP1 (pg/ml) as determined by ELISA comparing non-PDAC controls (n=58) with patients having PDAC (n=87) (p values determined by unpaired t-test). (N) Overall survival (OS) probability curve of patients with stage 1 PDAC from seven publicly available datasets analysed via OSpaad online software. At stage 1, the upper tercile of patients according to PGLYRP1 expression has poorer OS than the lower tercile. P-values determined by log-rank test. ANOVA, analysis of variance; ARG1, arginase1; CSCs, cancer stem cells; EpCAM, epithelial adhesion cell adhesion molecule; ITGAM, integrin subunit alpha M; MΦ, macrophage; MPO, heme protein myeloperoxidase; PDAC, pancreatic ductal adenocarcinoma; PDX, patient-derived xenograft; PGLYRP1, peptidoglycan recognition protein 1; PTPRC, protein tyrosine phosphatase receptor type-C; RT-qPCR, reverse-transcription quantitative PCR; scRNA-seq, single-cell RNA sequence; STDEV, standard deviation; TCGA, the cancer genome atlas; UMAP, Uniform Manifold Approximation and Projection; WT, wild type.

To validate the results obtained in PDAC murine models in human samples, we evaluated the expression of PGLYRP1 in patients. First, we assessed, by RNA-seq,^40^ the expression of *PGLYRP1* in EPCAM^+^ cells from the Espinet *et al* dataset.^40^ Overall, there was an increase in *PGLYRP1* expression in tumours compared with healthy adjacent tissue; however, many tumours expressed low levels of *PGLYRP1* (figure 8A), as would be expected of a marker (ie, CSC marker) whose expression is restricted to a subpopulation of cells. Thus, we analysed a scRNA-seq dataset obtained from primary tumours of patients with PDAC^41^ and found that PGLYRP1 was expressed in few cells, mainly in the tumour compartment (figure 8B,C). *PGLYRP1^+^* cells were enriched in genes related with stemness, aggressiveness and chemoresistance (figure 8D), in agreement with our results in KPC mice. We next validated the results obtained employing patient-derived xenograft (PDX)-derived primary PDAC cells (ie, PANC185 and PANC354) in vitro. As expected, human PDAC spheroid cultures expressed higher PGLYRP1 levels (figure 8E,F). Human PDAC cells are EPCAM^+^ but do not express Sca-1, as it is a murine protein with no human homologue. Thus, CD133 and CXCR4, which are markers linked with a metastatic CSC subpopulation,^4^ were used to verify the enrichment of PGLYRP1 expression in human CSCs ex vivo and in vitro (figure 8G,H). We also confirmed PGLYRP1 expression in epithelial tumour cells across a series of tissue samples including PDXs and freshly resected tumours and its absence in healthy tumour-adjacent pancreas (online supplemental figure S7A), with results similar to those obtained in our mouse models. To further validate these results, we analysed PGLYRP1 expression in a tissue microarray (TMA) containing cores from 113 patients. Although most of the patient samples expressed PGLYRP1 in the TME, a smaller fraction expressed PGLYRP1 in tumour cells (online supplemental figure S7B). Interestingly, PGLYRP1 expression was detected in 26.19% of samples with poor or undifferentiated histological tumour differentiation (grade 3–4) and in 11.94% with well to moderate differentiation (grade 1–2). Consequently, the grade 3–4 samples, associated with aggressive tumour behaviour, presented higher levels of PGLYRP1 than grade 1–2 samples, although it was not significant probably due to the limited number of samples (online supplemental figure S7C). Concerning immune evasion, we confirmed that PGLYRP1^+^ human cells also co-expressed PD-L1 and CD86 (figure 8I, online supplemental figure S7D). PGLYRP1 KO and OE cell lines were also generated (online supplemental figure S7E–G), and WT, OE and KO cells were cocultured with human MΦ and activated T cells to measure their capacity to avoid phagocytosis (figure 8J) and cytotoxic killing (figure 8K), confirming the immune evasive role of PGLYRP1 in the human setting. We also analysed immune infiltration ex vivo and in silico. In our TMA, patients with detectable levels of PGLYRP1 in the tumour compartment presented lower infiltration of immune cells (online supplemental figure S7H), although not significant, again probably due to the limited number of samples. Employing TCGA data, we found that *PGLYRP1* correlated negatively with CD45 and CD11b genes (*PTPRC* and *ITGAM*, respectively) and positively with *ARG1* and *MPO* (figure 8L).

To further study the interaction between human tumour cells and immune cells in vivo, we used a *Danio rerio* xenograft model, which has a functional innate immune system from birth.^42^ PDAC WT and KO cells were injected into 48-hour postfertilisation embryos, and tumour formation and metastatic capacity were measured up to 6 days postinjection (dpi). Although the growth rate of PANC185 cells was not suitable for quantification, PANC354 KO cells showed an impaired growth capacity compared with WT cells (online supplemental figure S7I), possibly due to immune clearance by MΦs. Concerning metastatic capacity, PGLYRP1 KO in both cell lines significantly reduced the number of tail micrometastases (online supplemental figure S7J,K), supporting the hypothesis that PGLYRP1 has a role in tumour formation and metastasis.

Next, we analysed the META,^43^ Moffitt,^44^ Janky^45^ and TCGA databases to additionally validate *PGLYRP1* expression in patient samples. The Moffitt dataset (GSE71729) revealed that 45 genes, including PGLYRP1, were commonly upregulated across primary tumour, liver and lung metastasis compared with healthy tissue (online supplemental figure S8A) and that most of the signalling pathways associated with these genes were related with the immune system and antibacterial defence (online supplemental figure S8B). We also interrogated PGLYRP1^high^ patients from the Janky, TCGA and META datasets, revealing downregulation of gene signatures such as inflammatory response or TNFα-signalling via nuclear factor kappa B (online supplemental figure S8C), highlighted in red). These findings demonstrate that PGLYRP1 is upregulated in PDAC and that immunomodulation of the TME appears to be a consistent feature associated with PGLYRP1 expression. Additionally, we explored a more general link between PGLYRP1 expression and CSC features by assessing PGLYRP1 expression in different cancer cell lines and observed an enrichment in PGLYRP1 expression in spheroids in all cell lines tested, except for colorectal cancer cells, suggesting that PGLYRP1 could also be important in CSCs of other tumour types, but it is not a pan-CSC marker (online supplemental figure S8D).

Finally, considering PGLYRP1 secretion, we assessed the potential utility of PGLYRP1 as a PDAC serum biomarker. 145 samples were analysed (58 non-PDAC controls, including 18 healthy controls, 25 high-risk controls, 19 patients with non-tumoral pancreatic diseases and 87 patients with PDAC), and PGLYRP1 levels were found to be significantly higher in patients with PDAC (figure 8M, online supplemental figure S8E), suggesting that PGLYRP1 could be a useful biomarker in differential PDAC diagnosis. Nevertheless, no differences in PGLYRP1 serum levels were found among different tumour stages (online supplemental figure S8F).

We also evaluated the relationship between PGLYRP1 expression and overall survival (OS) with Ospaad, an online tool published by Zhang *et al* which combines data from seven different patient cohorts (eg, TCGA and ICGC).^46^ Interestingly, patients at stage 1 could be stratified according to PGLYRP1 expression with the upper tercile exhibiting worse OS compared with the lower tercile (figure 8N). These data indicate that PGLYRP1 could potentially serve as a valuable and specific biomarker for detecting PDAC in liquid biopsies of patients and for stratifying patients at early stages in clinical settings, although more cohort studies are needed.

## Discussion

In this study, we identified and characterised a population of murine PDAC cells with CSC features in vitro and in vivo. Although EpCAM, Sca-1 and CD133 identified a stem-like population in vitro in normal pancreas, more assays are needed to validate them as *bona fide* stem cells. However, the three-marker combination identified a population of cells in PDAC tumours that expanded during tumour formation and possessed relevant CSC characteristics. Of note, EpCAM^+^CD24^+^CD44^+^CD133^-^Sca-1^-^ population bearing CSC properties and metastatic potential was reported in a KP^KO^C model.^47 48^ Additionally, two other works identified a CSC subpopulation in a KP^fl/fl^C mouse based on Musashi gene expression.^49 50^ These disparities may be due to variations in the CSC markers used and/or the lack and/or specific mutations affecting *Trp53* across the models. Moreover, Dosch *et al* showed that the KPC mouse model lacks ‘single marker-defined’ CSCs^51^; thus, a combination approach, such as the one we propose here with EpCAM, Sca-1 and CD133, will likely prove more accurate for identifying murine PDAC CSCs. Leinenkugel *et al* recently described that pancreatic cells with stem potential in both healthy and mutated *Kras* tumours were Sca-1^+^, which is in line with our results supporting the validity of Sca-1 as a potential marker for CSCs.^52^ While other marker combinations could also possibly detect PDAC CSCs, transcriptional and functional analyses validated our triple-positive population as *bona fide* CSCs. In addition to an enrichment in stem pathways, EpCAM^+^Sca-1^+^CD133^+^ cells also displayed a gene expression profile enriched in innate immunity and tumour immune evasion signatures.

Among the significantly upregulated genes, we identified *Pglyrp1*, which has a role in innate immunity as an antibacterial protein and participates as a proinflammatory factor in autoimmune diseases.^53 54^ Recently, it has been described as a protumour protein produced by T cells in some cancers.^32^ Furthermore, has been identified in the PDAC secretome.^37 55^ However, its functional role in PDAC had never been studied before. Here, we now present a novel role for PGLYRP1 as a critical contributor of CSC immune evasion in murine and human tumours. PGLYRP1^+^ cells displayed enhanced immune evasive properties, and its modulation could significantly impact these traits. For example, tumours generated by PGLYRP1 OE cells were characterised by decreased infiltration of immune cells, while the immune cell infiltration in mice pancreata injected with KO cells was significantly higher compared with healthy pancreata, suggesting that immune cells precluded tumour formation. The latter is supported by the fact that PGLYRP1 KO cells efficiently formed tumours in immunocompromised mice but not in immunocompetent animals, and in vitro, PGLYRP1 KO cells are more susceptible to T-cell-mediated killing and macrophage phagocytosis than WT and OE cells. Together, these data suggest that PGLYRP1 protects tumour cells, and in particular CSCs, from immune-mediated elimination. The latter is likely very necessary for the early steps of tumour formation to overcome the initial immune response and for the metastatic process. Indeed, PGLYRP1 was detectable in early PanIN lesions and in metastatic lesions. Furthermore, PGLYRP1 OE cells colonised more efficiently the liver than WT cells in our orthotopic models, and KO cells were incapable of metastatising in an orthotopic or intravenous injection model.

Regarding the protective mechanism of action of PGLYRP1, our results strongly point to an interaction between PGLYRP1 and TNFR1, as it appears that the interaction between them disrupts TNFα signalling and decreases cytotoxic susceptibility in PDAC cells. Inhibition of TNFα signalling with infliximab or rPGLYRP1 treatment led to protection against MΦ phagocytosis in PGLYRP1 KO cells. While more studies are required, PGLYRP1 certainly modulates the tumour immune cell composition and may play a previously unrecognised role in altering immune cell behaviour, promoting their protumoural characteristics and influencing their survival. Interestingly, we found that PGLYRP1 expression is induced via TNFα leading to immune evasion through several mechanisms. These results are in line with other works published in the last years. For example, Tekin *et al* described that MΦ-derived TNFα was cytotoxic for certain PDAC tumour cells,^56^ while recently Dixit *et al* showed that it induces immunosuppression by decreasing the expression of IL33.^39^ In addition, a study published by Tu *et al* associates TNFα with the ability to reprogram PDAC subtypes, inducing a more aggressive phenotype.^57^ Apart from these studies, Bianchi *et al* recently described how neutrophil-derived TNF regulates a tolerogenic circuit in PDAC via chemokine (C-X-C motif) ligand 1 (CXCL1).^58^ Likewise, and in agreement with these results, the analysis of human PDAC tumours carrying KRAS and TP53 alterations identified TNF signalling as a putative mediator of immunoregulation.^59^ Thus, TNFα signalling appears to be a key regulator of the PDAC immune status, as it drives the production of different molecules that directly affect the tumour immune landscape.

Although PDAC remains refractory to immune checkpoint inhibitors,^60^ some encouraging results are emerging in the field,^12 13^ suggesting that overcoming immunosuppression in PDAC will require targeting multiple pathways. Although TNFα targeting was presented as a promising therapy in PDAC to overcome its effects in immune evasion^61^ and tumour evolution,^57^ its application in clinical trials was not effective.^62 63^ Thus, targeting TNFα-related molecules instead might lead to more effective treatments as has been recently proposed.^64^ Since the available PGLYRP1 KO mouse is viable and only shows increased susceptibility to intraperitoneal infections,^65^ exploring the development of therapies to target PGLYRP1 as a potential immunotherapeutic approach should be considered. Furthermore, a recent article published by Schnell *et al* also pointed towards the potential of PGLYRP1 as a novel target for immunotherapy in cancer, as apart from reducing tumour size in other cancer models, its targeting did not generate autoimmune neuroinflammation.^32^ In addition, from a clinical point of view and supported by our results, PGLYRP1 could also be useful as a biomarker for patient stratification.

In summary, we have identified a subpopulation of CSCs that expresses PGLYRP1. Not only is PGLYRP1 a novel CSC marker, but it promotes immune evasive properties, plays a pivotal role in initiating pancreatic cancer and facilitates metastatic colonisation. Future investigations will elucidate the precise mechanisms underlying PGLYRP1-mediated immune evasion in pancreatic CSCs and its full clinical utility as a diagnostic marker and immunotherapeutic target for patients with PDAC.

## Materials and methods

### Primary and established cell line culture

Pancreatic tumours from KPC mice were harvested, cut into pieces and digested with collagenase P (Collagenase Type P, Cat no. J62406.03, Alfa Aesar) for 15 min at 37°C, followed by incubation with 0.25% trypsin for 3 min. Cells were then cultured in RPMI 1640 media (Invitrogen, Cat no. 61870044) containing 10% fetal bovine serum and 50 units/mL penicillin/streptomycin. Epithelial clones were picked, pooled and further expanded to obtain a heterogeneous cancer cell line. Human PANC185 and PANC354 PDAC PDX-derived cultures were established as previously described from PDXs.^66^ Original PDXs were obtained from Dr Manuel Hidalgo under a Material Transfer Agreement with the Spanish National Cancer Centre (CNIO), Madrid, Spain (reference no. I409181220BSMH). HEK293T, A549, Huh7, HN30, SW480, TTC-466 and HCC-1954 cell lines were cultured in RPMI 1640 media supplemented with 10% fetal bovine serum and 50 units/mL penicillin/streptomycin. Prior to use, all cell lines were authenticated and tested for mycoplasma contamination. For proliferation assays, 25 000 KPC ID11 or ID95 WT and PGLYRP1 KO cells were cultured, in triplicate, in multiwell plates in adherent conditions. Cells were trypsinised at 24, 48 and 72 hours later and counted, using 24 hours as a reference point.

### RNA expression microarrays

Expression arrays were performed at the CNIO Genomic Unit using the SurePrint G3 Mouse Gene Expression 8×60K arrays (Agilent Technologies) according to the manufacturer’s instructions. The microarray data have been deposited in GEO database with accession number ID: GSE222986. Images were quantified using Agilent Feature Extraction Software (V.10.1.1). Microarray background subtraction was carried out using the *normexp* method. Quantiles were used to perform normalisation. Filtered data were tested for differential expression applying R limma package^67^ (Bioconductor project; www.bioconductor.org). To account for testing of multiple hypotheses, the estimated significance level (p value) was adjusted using the Benjamini and Hochberg false discovery rate (FDR) correction. Those transcripts with an FDR <0.05 were selected as differentially expressed between the triple-positive population and all other tumour cells. Volcano plots were generated using GraphPad PRISM 8 representing -log(p value) versus log(fold change) of the differentially expressed genes between the triple-positive population and all other tumour cells. A heatmap was also generated with GraphPad PRISM 8. Normalised signal values for each gene were included for three KPC cell lines grown as spheroids compared with adherent monolayers.

GSEA was performed using annotations from Reactome, KEGG and additional gene sets from the Molecular Signature Database (MSigDB) (www.broadinstitute.org/gsea/index.jsp) or described in the literature.^59 68 69^ Detailed information about used gene sets is included in online supplemental table 1. Genes were ranked based on limma moderated t-statistics. After Kolmogorov-Smirnov testing, those gene sets showing an FDR of <0.15, a well-established cut-off for the identification of biologically relevant gene sets,^70^ were considered enriched between classes under comparison.

### In vivo tumourigenic assays

For orthotopic experiments, 10^4^ KPC ID11 or ID95 control, PGLYRP1 OE or KO cells were resuspended in 50 µL Matrigel and slowly injected into the pancreas of 10-week-old C57Bl/6J mice. Mice were sacrificed at 4 weeks postinjection. The pancreata, spleens, lungs and livers were excised, photographed, weighed and divided into two pieces. One of the pieces was fixed in 4% Paraformaldehyde (PFA) overnight at 4°C and subsequently paraffin embedded for histological analysis, while the other was digested as detailed above. Digested single-cell suspensions were used to determine the percentage of mCherry^+^ or BFP^+^ cells and the presence of micrometastases in the livers, as well as to determine the immune cell infiltration composition by flow cytometry using an Attune NxT Acoustic Cytometer (ThermoFisher Scientific). Antibodies used are detailed in online supplemental table 4. For tumourigenesis assays in NOD.CB17-Prkdc^scid/scid^/Rj mice (in-house breeding facility, Instituto de Investigaciones Biomedicas Sols-Morreale, CSIC-UAM), 10^3^ KPC ID11 or KPC ID95 control, OE or KO cells were resuspended in 50 µL Matrigel and subcutaneously injected into 10-week-old female mice and tracked for 3 weeks to confirm tumour formation. At the time of sacrifice, tumours were extracted, photographed, weighed and fixed in 4% PFA overnight at 4°C and subsequently paraffin embedded.

### Statistical analyses

Results are presented as means±SEM or standard deviation, as indicated. Student’s t-test was used to determine differences between means of groups unless otherwise stated. P<0.05 was considered statistically significant. Non-statistically significant results are displayed as ns. All analyses were performed using GraphPad Prism V.8.0 (San Diego California, USA).

### Data availability

Transcriptional data generated in this study from expression microarrays have been deposited in GEO database with accession number ID: GSE222986. Unique identifiers for publicly available datasets are indicated, a list of figures that have associated raw data can be provided and there are no restrictions on data availability.

Additional methods can be found in online supplemental methods.

## Data Availability

Data are available in a public, open access repository. Transcriptional data generated in this study from expression microarrays have been deposited in GEO database with accession number ID: GSE222986. Additional publicly available dataset references: Schlesinger Y, Yosefov-Levi O, Kolodkin-Gal D, Granit RZ, Peters L, Kalifa R, et al. Single-cell transcriptomes of pancreatic preinvasive lesions and cancer reveal acinar metaplastic cells’ heterogeneity. Nat Commun. 2020;11(1):4516.38. Nicolle R, Raffenne J, Paradis V, Couvelard A, de Reynies A, Blum Y, et al. Prognostic Biomarkers in Pancreatic Cancer: Avoiding Errata When Using the TCGA Dataset. Cancers. 2019;11(1).40. Espinet E, Gu Z, Imbusch CD, Giese NA, Büscher M, Safavi M, et al. Aggressive PDACs Show Hypomethylation of Repetitive Elements and the Execution of an Intrinsic IFN Program Linked to a Ductal Cell of Origin. Cancer discovery. 2021;11(3):638-59.41. Hwang WL, Jagadeesh KA, Guo JA, Hoffman HI, Yadollahpour P, Reeves JW, et al. Single-nucleus and spatial transcriptome profiling of pancreatic cancer identifies multicellular dynamics associated with neoadjuvant treatment. Nature genetics. 2022;54(8):1178-91.43. Martinelli P, Carrillo-de Santa Pau E, Cox T, Sainz B, Jr., Dusetti N, Greenhalf W, et al. GATA6 regulates EMT and tumour dissemination, and is a marker of response to adjuvant chemotherapy in pancreatic cancer. Gut. 2017;66(9):1665-76.44. Moffitt RA, Marayati R, Flate EL, Volmar KE, Loeza SG, Hoadley KA, et al. Virtual microdissection identifies distinct tumor- and stroma-specific subtypes of PDAC. Nature genetics. 2015;47(10):1168-78.45. Janky R, Binda MM, Allemeersch J, Van den Broeck A, Govaere O, Swinnen JV, et al. Prognostic relevance of molecular subtypes and master regulators in PDAC. BMC Cancer. 2016;16:632.
