# Correction: The Peptidoglycan Recognition Protein 1 confers immune evasive properties on pancreatic cancer stem cells

**DOI:** 10.1136/gutjnl-2023-330995corr1

**Published:** 2024-12-07

López-Gil JC, García-Silva S, Ruiz-Cañas L, et al. The Peptidoglycan Recognition Protein ^1^ confers immune evasive properties on pancreatic cancer stem cells. Gut 2024;73:1489-1508.

An additional affiliation for Dr Carmen Guerra should be:

Respiratory Tract Tumours Research Programme, Biomedical Research Network in Cancer (CIBERONC), Madrid, Spain

