# Supplementary material for: The Peptidoglycan Recognition Protein 1 confers immune evasive properties on pancreatic cancer stem cells
Source: Gut. 2024 May 16;73(9):1489–508. doi: 10.1136/gutjnl-2023-330995 (PMC11347225; doi:10.1136/gutjnl-2023-330995)
Supplement: Supplementary data [file gutjnl-2023-330995supp001.pdf]

BMJ Publishing Group Limited (BMJ) disclaims all liability and responsibility arising from any reliance placed on this supplemental material which has been supplied by the author(s)

The Peptidoglycan Recognition Protein 1 Confers Immune Evasive Properties on Pancreatic Cancer Stem Cells

Supplementary Figures

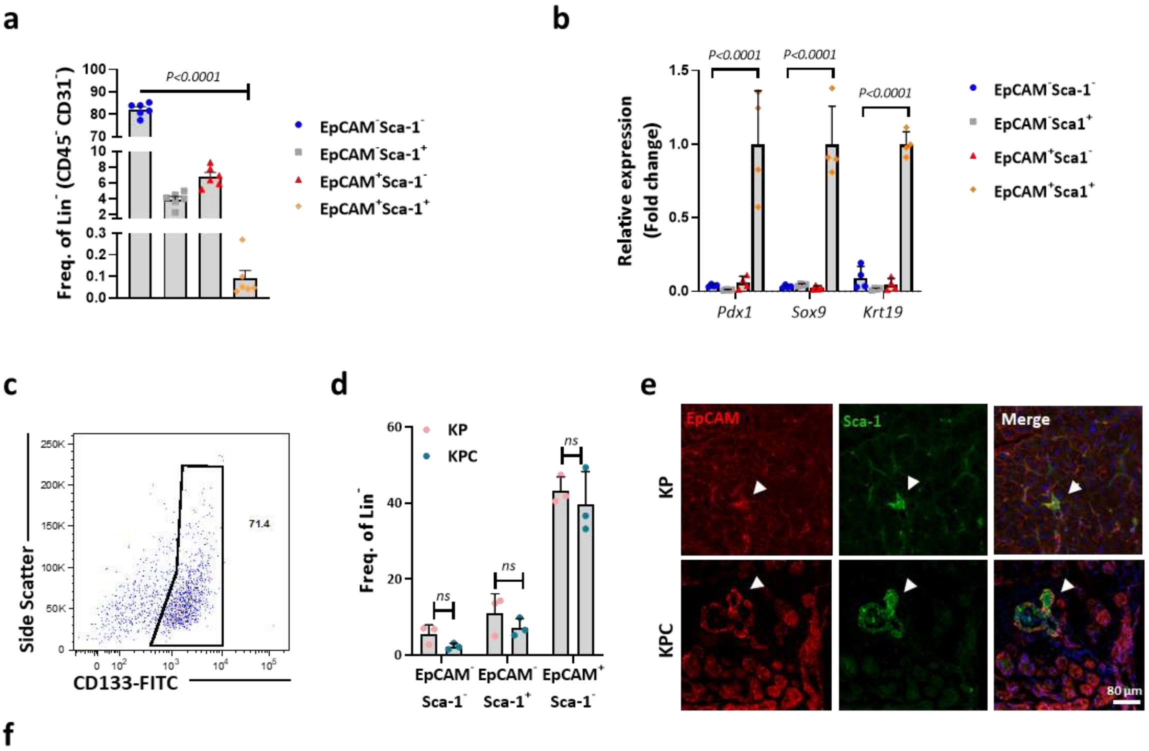

Number of cells	EpCAM⁻ Sca-1⁻	EpCAM⁻ Sca-1⁺	EpCAM⁺ Sca-1⁻
2.00E+04	0/4	3/4	1/4
1.00E+04	n.d.	0/13	1/13
1.00E+03	n.d.	0/2	0/2
1.00E+02	n.d.	0/4	0/4
**Freq. Of CSC**	<1.00E-05	<1.00E-05	<1.00E-05

Supplementary Figure 1. EpCAM⁺Sca1⁺ pancreatic cells retain stemness phenotypes. **(a)** Frequency (Freq) of different cell lineages (CD45 and CD31 negative populations) in the pancreas, defined by EpCAM and Sca-1 expression, shown as the mean ± SEM comparing each population with the double positive population (n=6, P values determined by One-Way ANOVA, with Dunnett's post-hoc test). Lin; Lineage. **(b)** RT-qPCR analysis of indicated markers for progenitor populations in the pancreas, shown as the mean fold change in mRNA levels ± STDEV with EpCAM⁺Sca-1⁺ set as 1.0 (n=4, P values determined by One-Way ANOVA, with Dunnett's post-hoc test). **(c)** Representative flow cytometry plot showing CD133 expression in EpCAM⁺Sca-1⁺ cells. **(d)** Differences in cell populations defined by EpCAM and Sca-1 expression in pancreata from KC and KPC mice at 8 weeks, shown as the mean of the frequency (Freq) ± STDEV (n=3, P values determined by Two-Way ANOVA test). **(e)** Representative confocal images of pancreata from 8-weeks old KP and KPC

animals simultaneously stained for EpCAM (red), Sca-1 (green) and DAPI (nuclear marker, blue). Arrowheads indicate double positive ($EpCAM^{+}Sca\text{-}1^{+}$) populations. Scale=80µm. **(f)** Panel detailing tumorigenic potential (number of tumors formed/number of injections) of the indicated FACS-sorted populations and predicted frequency (Freq.) of CSCs as a function of the dilutions tested. Indicated numbers of cells were resuspended in Matrigel™ and subcutaneously injected in different flanks of athymic nude mice.

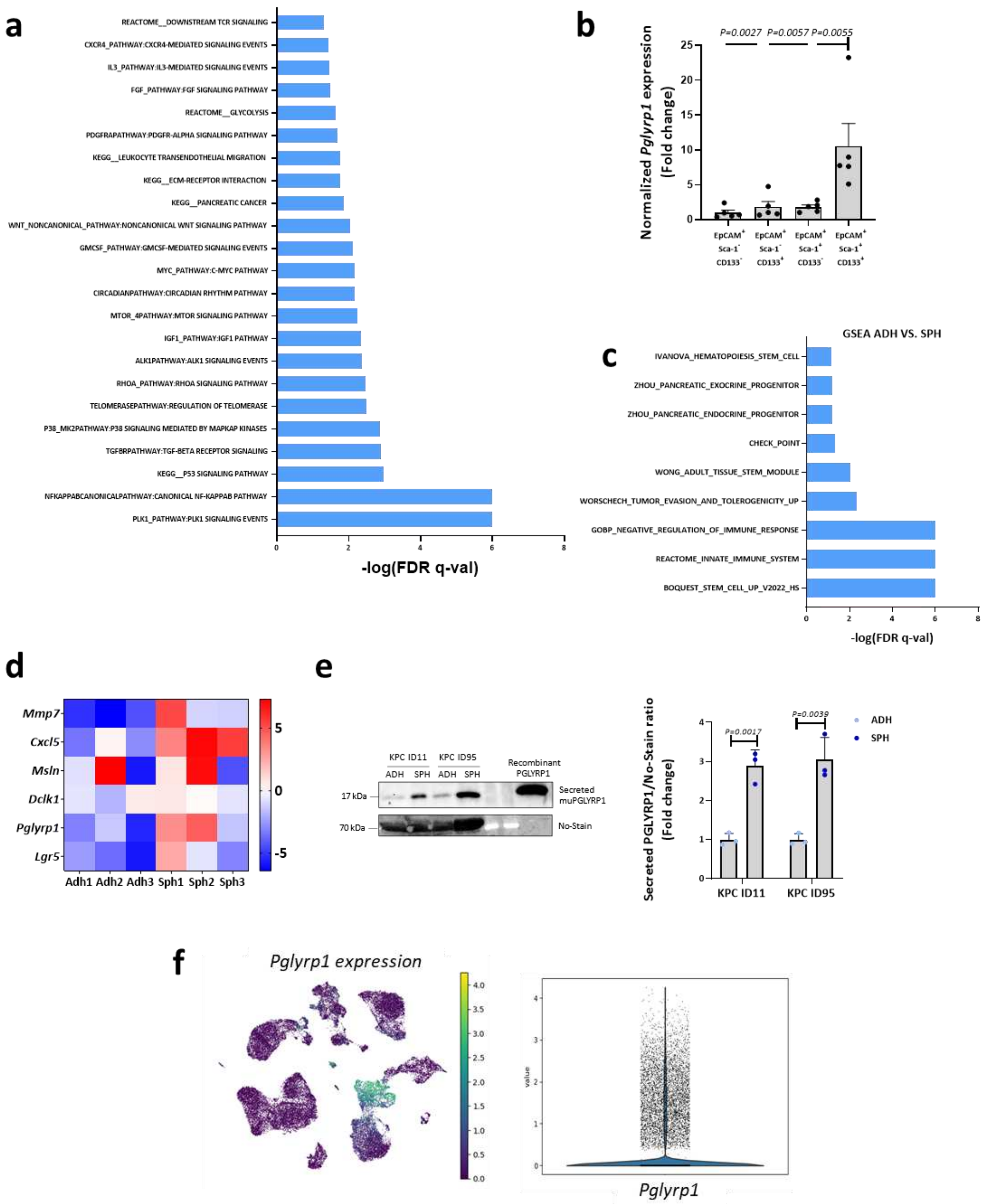

Supplementary Figure 2. CSC transcriptomic analysis, PGLYRP1 expression and secretion. (a) Gene sets enriched in the transcriptional profile of the triple-positive CSC population showing upregulation of pathways related with tumoral processes and innate immunity. Shown are the -log(FRD q-val) values for each pathway using the KEGG, NCI, REACTOME and custom signatures genesets, meeting the significance

criteria: nominal p-value of <0.05, FDR < 25%. **(b)** RT-qPCR analysis of *Pglyrp1* expression in the indicated sorted pancreatic tumor populations, shown as the mean fold change of *Pglyrp1* mRNA levels ± SEM (n=5, P values determined by One Way ANOVA, with Dunnett's post-hoc test). **(c)** Gene sets enriched in the transcriptional profile of murine tumor cells grown as spheroids (SPH) showing an upregulation in stem cell and immune evasion signatures compared with adherent (ADH) monolayers. Shown are the -log(FDR q-val) values for each pathway using REACTOME and custom signatures genesets, meeting the significance criteria: nominal p-value of <0.05, FDR < 25% (n=3 biological replicates). **(d)** Heat map for spheroids (Sph) versus adherent (Adh) monolayers showing the expression levels (z-score) of the genes previously identified to be enriched in triple-positive CSCs in (n=3 biological replicates). **(e)** Left panel: Representative Western Blot of secreted murine (mu) PGLYRP1 protein levels in conditioned media protein extracts derived from adherent (ADH) monolayer or spheroid (SPH) cultures. Recombinant muPGLYRP1 was included as a positive control, and the No-Stain Protein Labeling Reagent was included as a normalization control. Right panel: Densitometric quantification of secreted PGLYRP1 protein levels shown as the mean fold change of the PGLYRP1/No-Stain reagent ratio ± STDEV, with ADH set as 1.0 (n=3, P values determined by unpaired t-test). **(f)** Left panel: UMAP of *Pglyrp1* expression in the metaplastic compartment from the Schlesinger *et al.* dataset (1). Right panel: Violin plot indicating the level of expression in the metaplastic compartment.

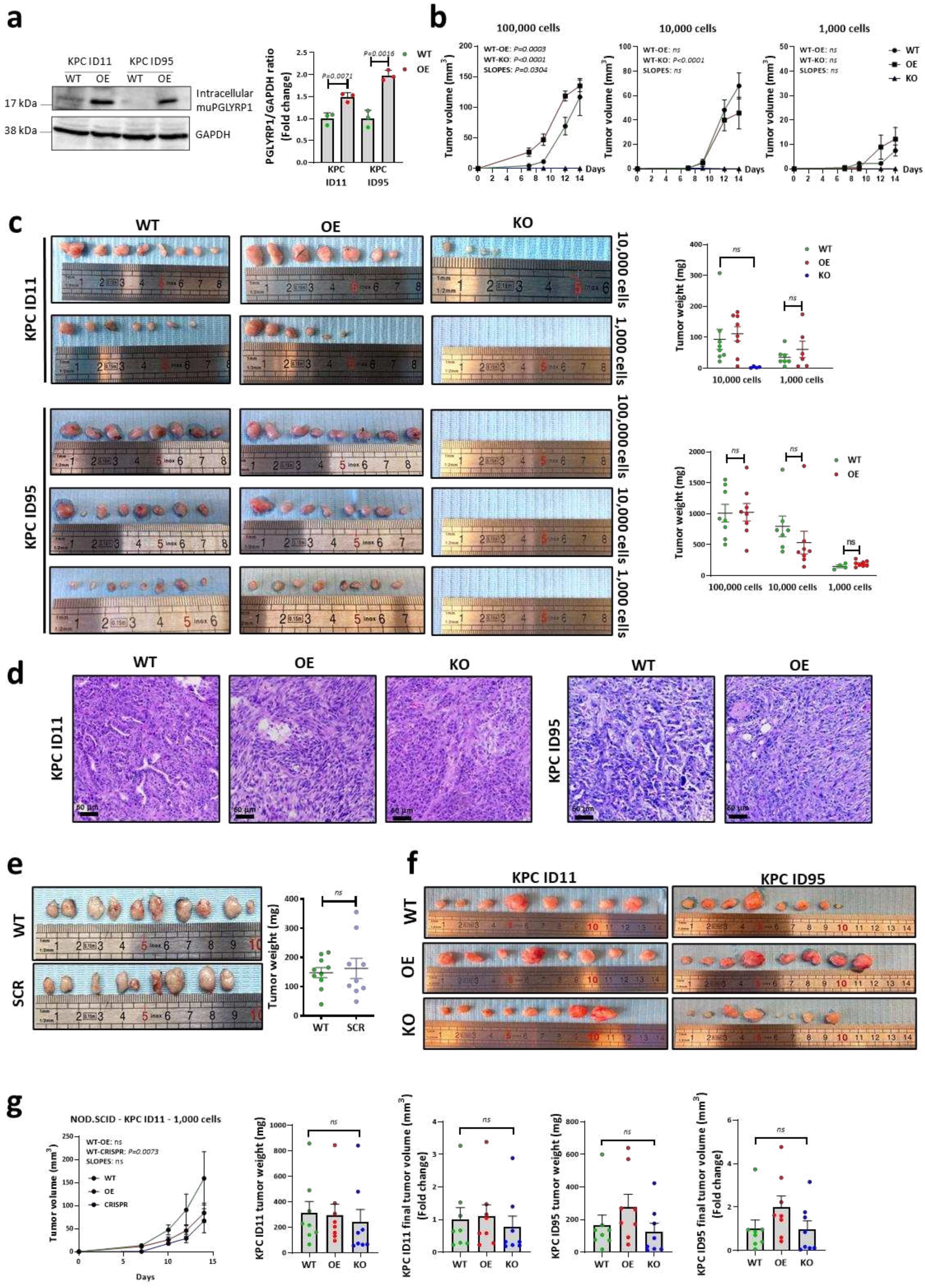
a
KPC ID11
KPC ID95
WT
OE
17 kDa
38 kDa
Intracellular muPGLYRP1
GAPDH
PGLYRP1/GAPDH ratio (Fold change)
P=0.0071
P=0.0016
b
100,000 cells
10,000 cells
1,000 cells
WT-OE: P=0.0003
WT-KO: P<0.0001
SLOPES: P=0.0304
Tumor volume (mm3)
Days
KO
c
KPC ID11
KPC ID95
10,000 cells
1,000 cells
100,000 cells
Tumor weight (mg)
ns
d
e
SCR
f
g
NOD.SCID - KPC ID11 - 1,000 cells
WT-CRISPR: P=0.0073
CRISPR
KPC ID11 tumor weight (mg)
KPC ID11 final tumor volume (mm3) (Fold change)
KPC ID95 tumor weight (mg)
KPC ID95 final tumor volume (mm3) (Fold change)

Supplementary Figure 3. PGLYRP1 is not essential for stemness. (a) Left panel: Western Blot analysis of intracellular murine (mu) PGLYRP1 expression in WT and OE cell lines (ID11 and ID95). GAPDH was used as a loading control. Right panel: Quantification of muPGLYRP1 expression in both cell lines, shown as the mean fold change in PGLYRP1/GAPDH densitometric levels ± STDEV, with WT set as 1.0 (n=3, P values determined by unpaired t-test). **(b)** Growth curves indicating the mean volume (mm^3) ± SEM over 14 days following injection of 100,000, 10,000 or 1,000 cells of ID95 wildtype (WT), overexpressing (OE) or KO cells (n of injections=8). The slopes for each group were compared using the "Comparing slopes tool" (GraphPad v8). P values to compare between groups were calculated by Two Way ANOVA. **(c)** Left panel: Images of tumors obtained after injection of the indicated number of WT, OE or KO cells (ID11 and ID95) in immunocompetent mice. Right panel: Quantification of the mean tumor weights (mg) ± SEM for WT, OE and/or KO tumors (n of injections=8, P values determined by One Way ANOVA, Tukey post-hoc test (ID11 10,000 cells) or unpaired t-test). **(d)** Representative H&E-stained sections of subcutaneous tumors formed from wildtype (WT), overexpressing (OE) or KO cells injected in immunocompetent mice for the above-described ELDA. Scale bars = 60 µm. **(e)** Left panel: Images of tumors obtained after injection of 100,000 WT or Scramble/Cas9 (SCR) KPC cells in immunocompetent mice. Right panel: Quantification of the mean tumor weights (mg) ± SEM for WT and SCR tumors (n of injections=10, P values determined by unpaired t-test). **(f)** Images of tumors obtained after injection of 1,000 WT, OE or KO cells (ID11 and ID95) in NOD.SCID mice. **(g)** Left panel: Growth curves indicating the mean volume (mm^3) ± SEM over 14 days following injection of 1,000 cells of ID11 WT, OE or KO cells in NOD.SCID mice (n of injections=8). The slopes for each group were compared using the "Comparing slopes tool" (GraphPad v8). P values to compare between groups were calculated by Two Way ANOVA. Right panels: Quantification of the mean tumor weights (mg) or mean final tumor volumes (mm^3) ± SEM for WT, OE and/or KO tumors. Volumes shown as the mean fold change with WT set as 1.0 (n of injections=8, P values determined by One Way ANOVA, with Tukey post-hoc test).

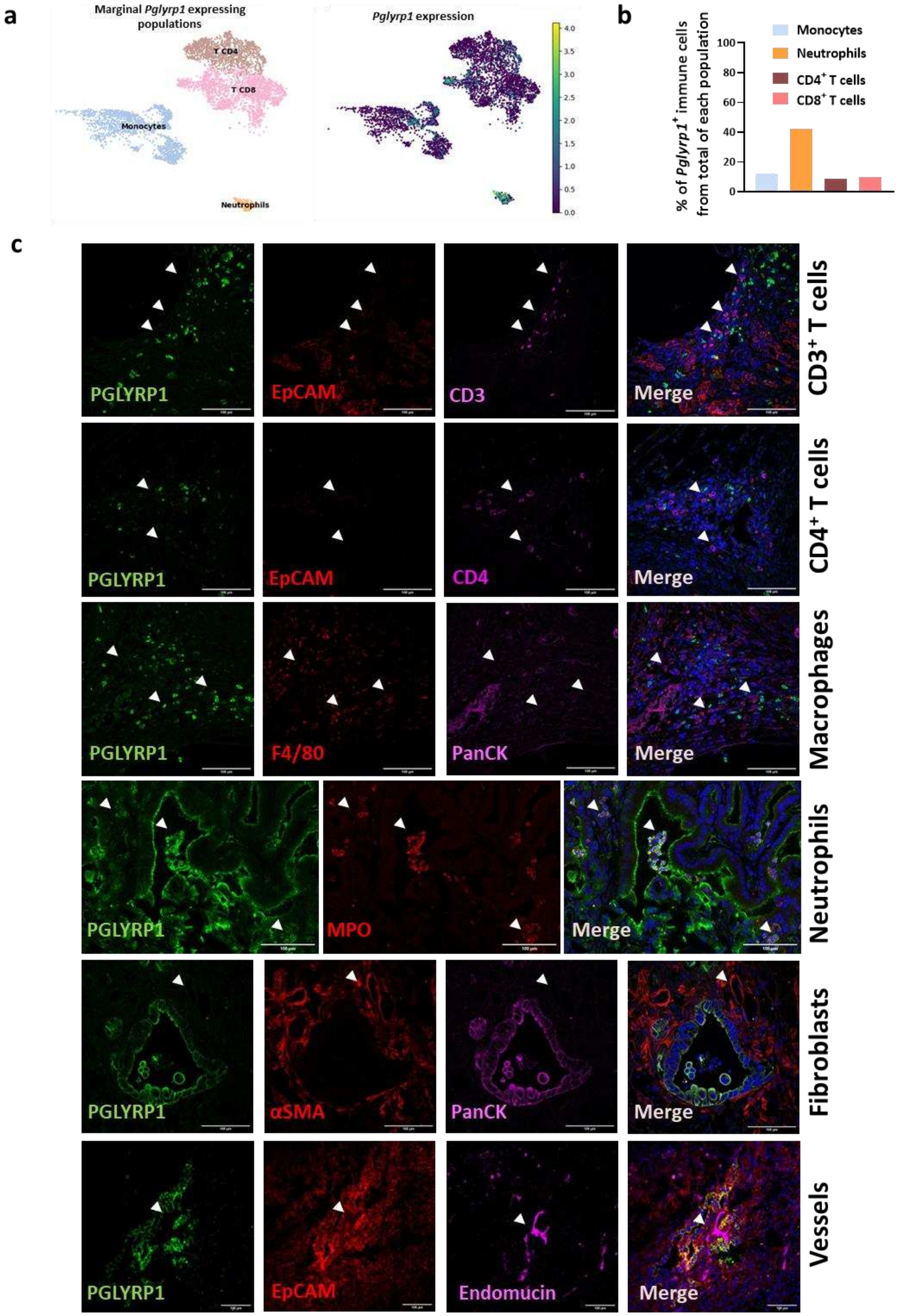
a
Marginal *Pglyrp1* expressing populations
T CD4
T CD8
Monocytes
Neutrophils
Pglyrp1 expression
4.0
3.5
3.0
2.5
2.0
1.5
1.0
0.5
0.0
b
% of *Pglyrp1*+ immune cells from total of each population
100
80
60
40
20
0
Monocytes
Neutrophils
CD4+ T cells
CD8+ T cells
c
PGLYRP1
EpCAM
CD3
Merge
CD3+ T cells
PGLYRP1
EpCAM
CD4
Merge
CD4+ T cells
PGLYRP1
F4/80
PanCK
Merge
Macrophages
PGLYRP1
MPO
Merge
Neutrophils
PGLYRP1
αSMA
PanCK
Merge
Fibroblasts
PGLYRP1
EpCAM
Endomucin
Merge
Vessels

Supplementary Figure 4. PGLYRP1 expression on stroma cells. **(a)** UMAPs of the immune populations in murine tumors which express *Pglyrp1*, being present in neutrophils and some subsets of monocytes and T cells. **(b)** Quantification of the frequency in the percentage of *Pglyrp1* expressing immune cells from the total population. **(c)** Representative confocal microscopy images of KPC tumors stained for PGLYRP1 (green), tumor cell markers (EpCAM or PanCK), immune cell markers (CD3, CD4, F4/80 or MPO), fibroblasts (αSMA) or vessels markers (Endomucin). Arrowheads indicate PGLYRP1^{+} immune cells in the tumor microenvironment. Scale bars = 100 µm.

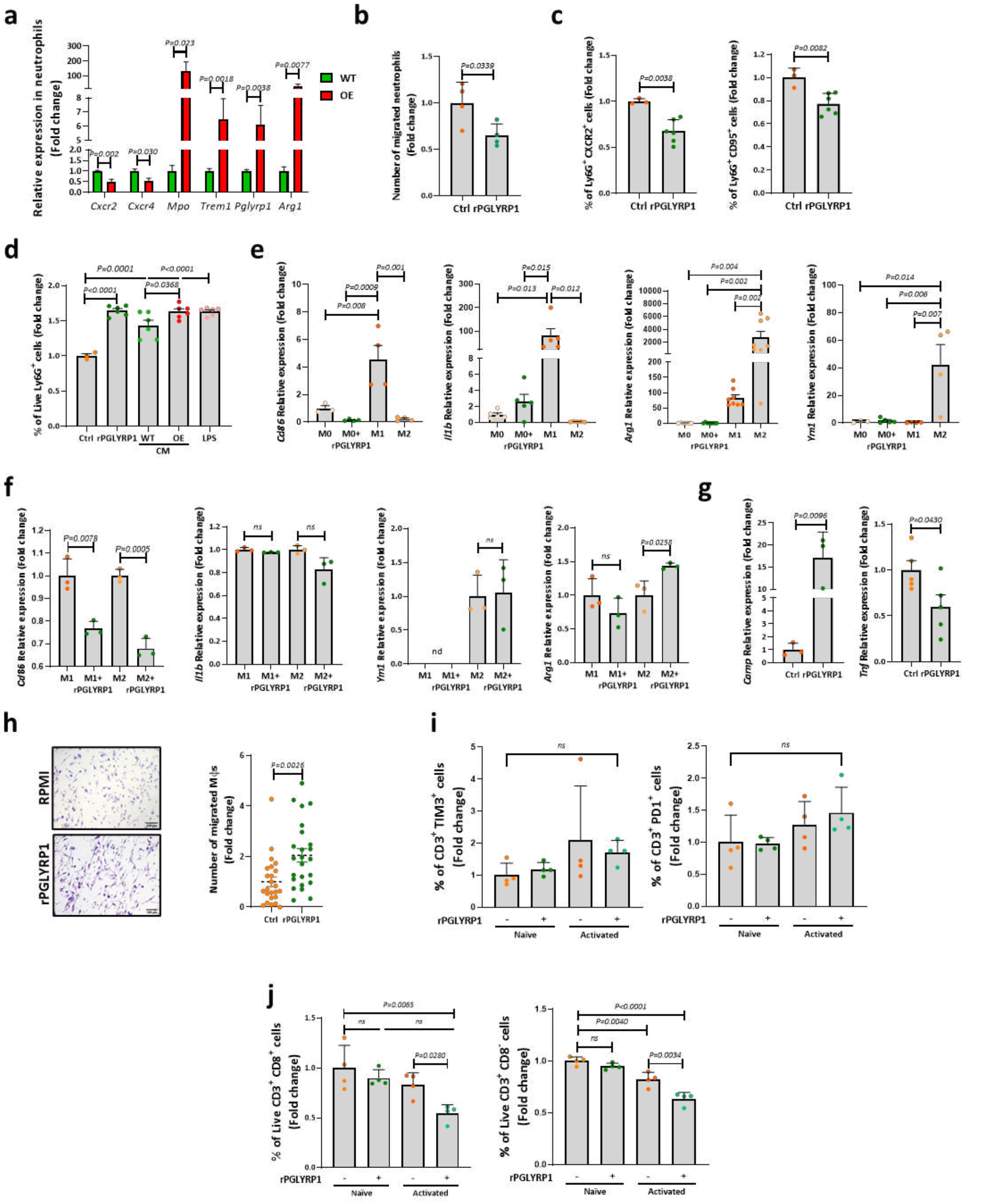

Supplementary Figure 5. The effects of PGLYRP1 on neutrophils, macrophages and T cells. (a) RT-qPCR analysis of genes related with migration and immune evasion in sorted neutrophils from KPC PGLYRP1 WT and OE tumors, shown as the mean fold change in mRNA levels ± SEM, with WT set as 1.0 (n=3 tumors, P

values determined by unpaired t-test). **(b)** Quantification of the number of migrated freshly-isolated neutrophils exposed to RPMI (Ctrl) or RPMI+rPGLYRP1 (rPGLYRP1) in a trans-well system, represented as mean fold change ± STDEV with Ctrl set as 1.0 (n=4, P values determined by unpaired t-test). Neutrophils were obtained from spleens from 3 different mice. **(c)** Quantification of flow cytometric analysis of the percentage of Ly6G$^+$/CXCR2$^+$ or Ly6G$^+$/CD95$^+$ freshly isolated neutrophils after rPGLYRP1 exposure, represented as mean fold change ± STDEV with Ctrl set as 1.0 (n=3 for controls and n=6 for rPGLYRP1, P values determined by unpaired t-test). Neutrophils were obtained from spleens from 3 different mice. **(d)** Quantification of live neutrophils, by flow cytometry, after treatment with LPS, rPGLYRP1, KPC WT- or KPC OE-derived conditioned medium (CM). Shown is the mean fold change ± SEM, with Ctrl set as 1.0 (n=3 for RPMI group and n=6 for the rest, P values determined by One Way ANOVA, with Tukey post-hoc test). **(e)** RT-qPCR analysis of genes related with MΦ polarization in untreated (M0), M1-like, M2-like, or rPGLYRP1-treated non-polarized MΦs, shown as the mean fold change in mRNA levels ± STDEV, with M0 set as 1.0 (n=4, P values determined by One Way ANOVA, with Dunnett's post-hoc test). **(f)** RT-qPCR analysis of the previously mentioned genes in polarized MΦs with or without rPGLYRP1, shown as the mean fold change in mRNA levels ± STDEV, with untreated MΦs set as 1.0 (n=3, P values determined by unpaired t-test). **(g)** RT-qPCR analysis of *Camp* and *Tnf* expression in untreated (Ctrl) or rPGLYRP1-treated non-polarized MΦs, shown as the mean fold change in *Camp* or *Tnf* mRNA levels ± STDEV, with Ctrl set as 1.0 (n=3, P values determined by unpaired t-test). **(h)** Left panel: Pictures of crystal violet stained migrated MΦs in transwell inserts with RPMI or RPMI plus rPGLYRP1 (n=3). Scale=100µm. Right panel: Number of migrated MΦs exposed to RPMI (Ctrl) or RPMI+rPGLYRP1 (rPGLYRP1) in a trans-well system, shown as the mean fold change ± SEM with Ctrl set as 1.0 (n=24, P values determined by unpaired t-test). **(i)** Left panel: Quantification of TIM3$^+$ activated or non-activated (naïve) live (DAPI$^-$) CD3$^+$ T cells after rPGLYRP1 treatment. Shown is the mean fold change ± STDEV, with naïve non-treated cells (control) set as 1.0 (n=4, P values determined by Two Way ANOVA). Right panel: Quantification of PD1$^+$ activated or non-activated (naïve) live (DAPI$^-$) CD3$^+$ T cells after rPGLYRP1 treatment. Shown is the mean fold change ± STDEV, with naïve non-treated cells (control) set as 1.0 (n=4, P values determined by Two Way ANOVA). **(j)** Left panel: Quantification of activated

or non-activated (naïve) live ($DAPI^{-}$) $CD3^{+}CD8^{+}$ T cells after rPGLYRP1 treatment. Shown is the mean fold change ± STDEV, with naïve non-treated cells (control) set as 1.0 (n=4, P values as determined by Two Way ANOVA). Right panel: Quantification of activated or non-activated (naïve) live ($DAPI^{-}$) $CD3^{+}CD8^{-}$ T cells after rPGLYRP1 treatment. Shown is the mean fold change ± STDEV, with naïve non-treated cells (control) set as 1.0 (n=4, P values determined by Two Way ANOVA).

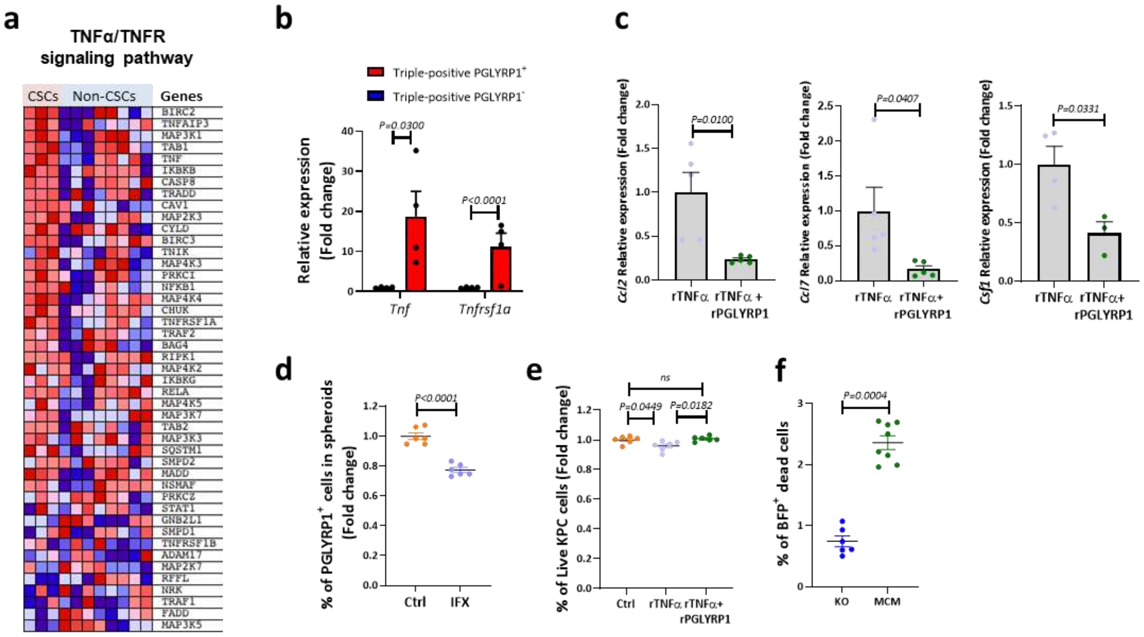

Supplementary Figure 6. PGLYRP1 counteracts TNFα by blocking its effects *in vitro*. (a) Heatmap showing the upregulated genes from the TNF-TNFR signaling pathway in triple-positive CSCs versus non-CSCs (n=3 for each population). **(b)** RT-qPCR analysis of *Tnf* and *Tnfrsf1a* expression in triple-positive PGLYRP1$^+$ or PGLYRP1$^-$ CSCs, shown as the mean fold change in mRNA levels ± SEM, with triple-positive PGLYRP1$^+$ cells set as 1.0 (n=4, P values determined by unpaired t-test). **(c)** RT-qPCR analysis of downstream genes regulated by TNFα-TNFR1 signaling in KPC cells after recombinant (r)TNFα and rTNFα+rPGLYRP1 treatment for 6 hours, shown as the mean fold change in mRNA levels ± SEM, with rTNFα treated cells set as 1.0 (n=3, P values determined by unpaired t-test). **(d)** Quantification of the percentage of PGLYRP1$^+$ KPC cells grown as spheroids in control (Ctrl) and Infliximab-treated (IFX) conditions, shown as the mean fold change ± SEM with Ctrl set as 1.0 (n=6, P values determined by unpaired t-test). **(e)** Quantification by flow cytometry of live cells after rTNFα and rTNFα+rPGLYRP1 treatment for 6 hours, represented as the mean fold change ± SEM, with control set as 1.0 (n=6, P values determined by unpaired t-test). **(f)** Quantification by flow cytometry of BFP$^+$ PGLYRP1 KO dead cells after MΦ-conditioned media (MCM) treatment for 24 hours, represented as the mean fold change ± SEM, with KO set as 1.0 (n=6 for KO and n=8 for MCM, P values determined by unpaired t-test).

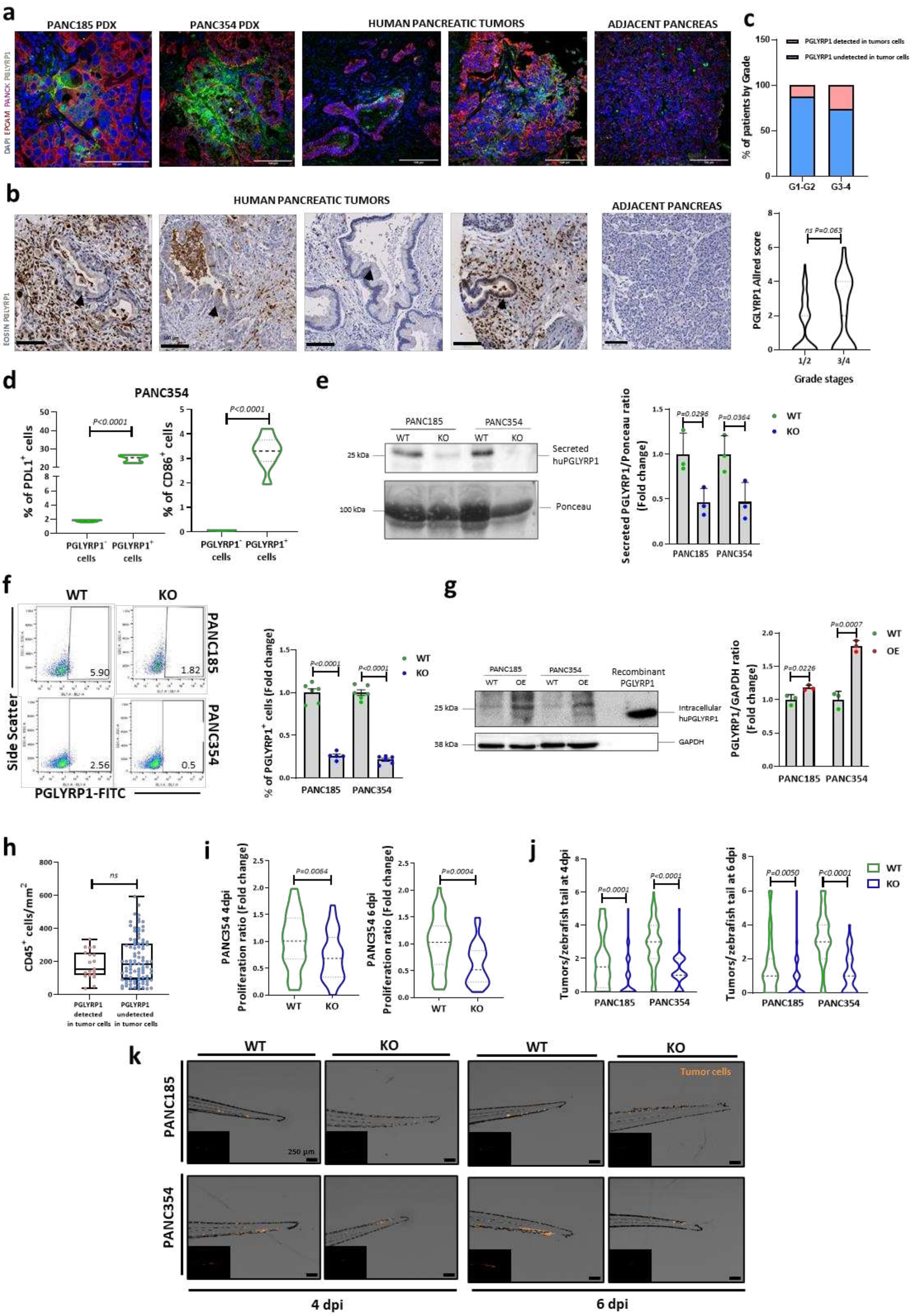
a
PANC185 PDX
PANC354 PDX
HUMAN PANCREATIC TUMORS
ADJACENT PANCREAS
DAPI EPCAM PANCK PGLYRP1
c
PGLYRP1 detected in tumors cells
PGLYRP1 undetected in tumor cells
% of patients by Grade
G1-G2
G3-4
b
HUMAN PANCREATIC TUMORS
ADJACENT PANCREAS
EOSIN PGLYRP1
ns P=0.063
PGLYRP1 Allred score
Grade stages
d
PANC354
P<0.0001
% of PDL1+ cells
% of CD86+ cells
PGLYRP1- cells
PGLYRP1+ cells
e
PANC185
PANC354
WT
KO
25 kDa
100 kDa
Secreted huPGLYRP1
Ponceau
P=0.0296
P=0.0364
Secreted PGLYRP1/Ponceau ratio (Fold change)
f
WT
KO
PANC185
PANC354
Side Scatter
PGLYRP1-FITC
5.90
1.82
2.56
0.5
P<0.0001
% of PGLYRP1+ cells (Fold change)
g
OE
Recombinant PGLYRP1
Intracellular huPGLYRP1
GAPDH
38 kDa
P=0.0226
P=0.0007
PGLYRP1/GAPDH ratio (Fold change)
h
ns
CD45+ cells/mm2
PGLYRP1 detected in tumor cells
PGLYRP1 undetected in tumor cells
i
P=0.0064
P=0.0004
PANC354 4dpi Proliferation ratio (Fold change)
PANC354 6 dpi Proliferation ratio (Fold change)
j
P=0.0001
P<0.0001
P=0.0050
Tumors/zebrafish tail at 4dpi
Tumors/zebrafish tail at 6 dpi
k
Tumor cells
250 µm
4 dpi
6 dpi

Supplementary Figure 7. Effects of PGLYRP1 modulation in human PDAC cells *in vitro* and *in vivo*. (a) Representative immunofluorescence confocal-microscopy images of PGLYRP1 (green), EPCAM (red), PANCK (pink) and DAPI (nuclear marker, blue) expression in human PDXs (PANC185 and PANC354), human PDAC tumors and healthy adjacent human pancreatic tissue. Scale bars = 100 µm. **(b)** Representative images of human PDAC tumor tissue and healthy adjacent tissue included in a tissue microarray and stained for PGLYRP1 (DAB, brown). PGLYRP1 was detected in immune cells present in the stroma and in epithelial tumor cells (indicated with black arrowheads). Scale=100µm. **(c)** Top panel: Percentage of patients with PGLYRP1 expression in tumor cells (pink) and with undetected PGLYRP1 expression (blue) segmented by grade. Bottom panel: Frequency of patients classified by grade according to PGLYRP1 detection in tumor cells and total PGLYRP1 expression quantified by semiquantitative Allred score in patients segmented by grade (n=113, P values determined by unpaired t-test). **(d)** Violin plots representing the mean percentage of $PDL1^+$ or $CD86^+$ cells in the $PGLYRP1^-$ or $^+$ populations in PANC354 (n=6 for PDL1 and n=9 for CD86, P values determined by unpaired t-test). **(e)** Left panel: Representative Western Blot of secreted human (hu) PGLYRP1 in conditioned media protein samples from wildtype (WT) and KO PANC185 and PANC354 primary PDX-derived cultures. Ponceau staining was included as a normalization control. Right panel: Densitometric quantification represented as the mean fold change of PGLYRP1/Ponceau ratio ± STDEV, with WT set as 1.0 (n=3, P values determined by unpaired t-test). **(f)** Left panel: Representative flow cytometry plots of the percentage of $PGLYRP1^+$ cells in wildtype (WT) and KO PANC185 and PANC354 primary PDX-derived cultures. Right panel: Quantification represented as the mean fold change in PGLYRP1 expression ± SEM, with WT set as 1.0 (n=6 P values determined by unpaired t-test). **(g)** Left panel: Representative Western Blot analysis of intracellular human (hu) PGLYRP1 expression in PANC185 and PANC354 cells: WT control and overexpressing (OE) PGLYRP1. GAPDH was included as a loading control. Right panel: Densitometric quantification represented as the mean fold change in PGLYRP1/GAPDH ratio ± STDEV, with WT set as 1.0 (n=3, P values determined by unpaired t-test). **(h)** Quantification of $CD45^+$ cells per mm^2 of tumor in the tissue microarray with samples stratified by detectable or undetectable staining for PGLYRP1 in tumor cells (n= 113 patients, P values determined by unpaired t-test). **(i)** Quantification of the proliferation of PANC354 WT and KO cells

at 4- and 6-days post injection (dpi) compared to 1 dpi in a *D. renio* zebrafish xenograft model. Shown are violin plots representing the mean fold change in proliferation, with WT set as 1.0 (n=37, P values determined by unpaired t-test). **(j)** Quantification of the number of tumors present in the tail of each zebrafish injected with WT or KO cells at 4 and 6 dpi. Using the GraphPad function 'Identify Outliers', outliers were discarded and afterwards an unpaired t-test was used with a confidence interval of 95% to compare the different conditions. **(k)** Representative images of zebrafish embryos xenografted with PANC185 and PANC354 WT or PGLYRP1 KO cells at 4 and 6 dpi. Images are shown in gray scale merged with fluorescent images of the injected cells. Fluorescence channel alone is added to each image for better visualization of the cells and the distribution (left bottom inlet). Scale bar = 250µm.

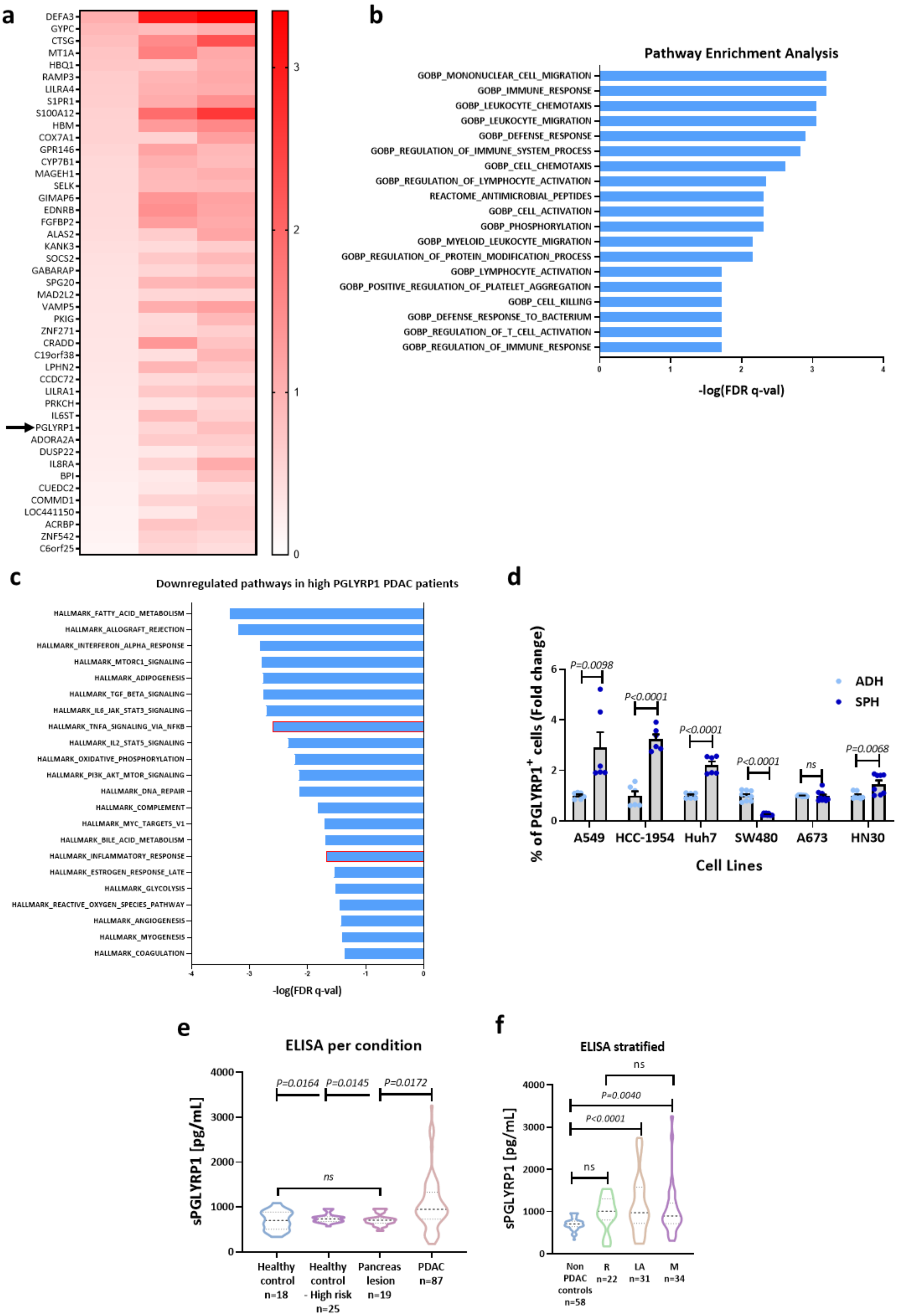
a
DEFA3
GYPC
CTSG
MT1A
HBQ1
RAMP3
LILRA4
S1PR1
S100A12
HBM
COX7A1
GPR146
CYP7B1
MAGEH1
SELK
GIMAP6
EDNRB
FGFBP2
ALAS2
KANK3
SOCS2
GABARAP
SPG20
MAD2L2
VAMP5
PKIG
ZNF271
CRADD
C19orf38
LPHN2
CCDC72
LILRA1
PRKCH
IL6ST
PGLYRP1
ADORA2A
DUSP22
IL8RA
BPI
CUEDC2
COMMD1
LOC441150
ACRBP
ZNF542
C6orf25
b
Pathway Enrichment Analysis
GOBP_MONONUCLEAR_CELL_MIGRATION
GOBP_IMMUNE_RESPONSE
GOBP_LEUKOCYTE_CHEMOTAXIS
GOBP_LEUKOCYTE_MIGRATION
GOBP_DEFENSE_RESPONSE
GOBP_REGULATION_OF_IMMUNE_SYSTEM_PROCESS
GOBP_CELL_CHEMOTAXIS
GOBP_REGULATION_OF_LYMPHOCYTE_ACTIVATION
REACTOME_ANTIMICROBIAL_PEPTIDES
GOBP_CELL_ACTIVATION
GOBP_PHOSPHORYLATION
GOBP_MYELOID_LEUKOCYTE_MIGRATION
GOBP_REGULATION_OF_PROTEIN_MODIFICATION_PROCESS
GOBP_LYMPHOCYTE_ACTIVATION
GOBP_POSITIVE_REGULATION_OF_PLATELET_AGGREGATION
GOBP_CELL_KILLING
GOBP_DEFENSE_RESPONSE_TO_BACTERIUM
GOBP_REGULATION_OF_T_CELL_ACTIVATION
GOBP_REGULATION_OF_IMMUNE_RESPONSE
-log(FDR q-val)
c
Downregulated pathways in high PGLYRP1 PDAC patients
HALLMARK_FATTY_ACID_METABOLISM
HALLMARK_ALLOGRAFT_REJECTION
HALLMARK_INTERFERON_ALPHA_RESPONSE
HALLMARK_MTORC1_SIGNALING
HALLMARK_ADIPOGENESIS
HALLMARK_TGF_BETA_SIGNALING
HALLMARK_IL6_JAK_STAT3_SIGNALING
HALLMARK_TNFA_SIGNALING_VIA_NFKB
HALLMARK_IL2_STAT5_SIGNALING
HALLMARK_OXIDATIVE_PHOSPHORYLATION
HALLMARK_PI3K_AKT_MTOR_SIGNALING
HALLMARK_DNA_REPAIR
HALLMARK_COMPLEMENT
HALLMARK_MYC_TARGETS_V1
HALLMARK_BILE_ACID_METABOLISM
HALLMARK_INFLAMMATORY_RESPONSE
HALLMARK_ESTROGEN_RESPONSE_LATE
HALLMARK_GLYCOLYSIS
HALLMARK_REACTIVE_OXYGEN_SPECIES_PATHWAY
HALLMARK_ANGIOGENESIS
HALLMARK_MYOGENESIS
HALLMARK_COAGULATION
-log(FDR q-val)
d
% of PGLYRP1+ cells (Fold change)
P=0.0098
P<0.0001
P<0.0001
P<0.0001
ns
P=0.0068
ADH
SPH
A549
HCC-1954
Huh7
SW480
A673
HN30
Cell Lines
e
ELISA per condition
sPGLYRP1 [pg/mL]
P=0.0164
P=0.0145
P=0.0172
ns
Healthy control n=18
Healthy control - High risk n=25
Pancreas lesion n=19
PDAC n=87
f
ELISA stratified
sPGLYRP1 [pg/mL]
ns
P=0.0040
P<0.0001
ns
Non PDAC controls n=58
R n=22
LA n=31
M n=34

Supplementary Figure 8. Analyses of human PDAC samples and other tumors. (a) Heatmap representing common upregulated genes for PDAC primary tumors, and liver and lung metastases compared to their healthy counterparts obtained from the Moffitt database. *PGLYRP1* is indicated with an arrow. **(b)** Common downregulated pathways in patients with high *PGLYRP1* levels from the TCGA, Janky and META databases. Shown are the -log(FRD q-val) values for each pathway geneset used, meeting the significance criteria: nominal p-value of <0.05, FDR < 25%. **(c)** Pathway enrichment analysis obtained from the common upregulated genes from the Moffitt database. Shown are the -log(FRD q-val) values for each pathway geneset used, meeting the significance criteria: nominal p-value of <0.05, FDR < 25%. Mentioned pathways are highlighted in red. **(d)** Flow cytometric analysis of the percentage of PGLYRP1^{+} cells in spheroids (SPH) and adherent (ADH) cultures from the indicated different tumor cell lines, shown as the mean fold change ± SEM, with ADH set as 1.0 (n=3, P values determined by unpaired t-test). A549, lung adenocarcinoma cells; HCC-1954, breast cancer cells; Huh7, hepatocellular carcinoma cells; SW480, colorectal cancer cells; A673, Ewing's sarcoma cells; and HN30, head and neck squamous carcinoma cells. **(e)** Violin plots representing the mean levels of soluble PGLYRP1 (pg/ml) as determined with an ELISA assay in healthy donors, healthy donors with high risk of developing PDAC, patients with other pancreatic lesions and PDAC patients (P values determined by One-Way ANOVA, Tukey post-hoc test). **(f)** Violin plots representing the mean levels of soluble PGLYRP1 (pg/ml) as determined with an ELISA assay. Non-PDAC controls (n=58) versus indicated stages (R: resectable, LA: locally advanced, M: metastasis) (P values determined by One-Way ANOVA, Tukey post-hoc test).

The Peptidoglycan Recognition Protein 1 Confers Immune Evasive Properties on Pancreatic Cancer Stem Cells

Supplementary Methods

Database interrogation and analyses. Correlations between human genes present in the TCGA dataset were made applying a Pearson r correlation. The Moffit database (GSE71729) was analyzed using NCBI's GEO2R, clustering and comparing samples in 3 categories (healthy pancreas vs. primary tumor, healthy liver vs. liver PDAC metastases, healthy lung vs. lung PDAC metastases). P-value was adjusted by the Benjamini and Hochberg method, and significantly ($p<0.05$) up- and downregulated genes were analyzed. A heatmap was made using GraphPad PRISM 8. Gene set enrichment analysis (GSEA) was performed using the up- and down-regulated genes from the Moffit database and comparing the patient samples expressing high levels of PGLYRP1 to those expressing low levels of PGLYRP1 from the TCGA, META and Bailey datasets (2-4).

OS curves were made using OSpaad (5) by selecting the combination of all the seven datasets available and comparing the upper tercile of patients according to *PGLYRP1* expression versus the lower tercile of stage I or stage III PDAC patients.

Murine scRNA-seq dataset (GSE141017) was downloaded from the Gene Expression Omnibus (GEO) database, corresponding to the work of Schlesinger et al., 2020. Data consists of six-to-eight weeks old Ptf1a-Cre^{ER}, LSL-Kras^{G12D}, LSL-tdTomato (PRT) mice from which pancreatic tissue was collected for single-cell isolation at six different time points post-Tamoxifen injection (PTI): Two mice from 3 months PTI, two mice from 5 months PTI and one mouse for each of the other time points: 17 days, 6 weeks and 9 months PTI. While almost all late-stage samples include noninvasive low-grade PanINs, a 15 months PTI mouse was also sampled, which did develop an invasive PDAC. Initially available filtered raw transcript counts formatted as gene-cell matrices were imported into Scanpy (v.1.7.2) via Python (6). Transcripts were filtered to remove samples with less than 200 counts per cell among all genes and with a mitochondrial gene content higher than 20%. Genes or features expressed in less than three cells were also excluded from the analysis. Finally, doublets were removed from the dataset using the Solo tool form scVI tools (v.0.19.0). Scanpy was used for data normalization, log-transformation, selection of highly variable genes and principal component analysis (PCA). Normalization of expression levels was carried out at a count level, to counts per 10000; and was then followed by log2 transformation. Log-transformed data was used for the identification of highly variable genes using the Scanpy Wilcoxon test function ranked_genes_group(), selecting those with adj <0.05 and |Log2foldchange| > 1, which

were then subjected to PCA. The optimal number of principal components was calculated using the Elbow fit method. PCA results were visualized via Uniform Manifold Approximation and Projection (UMAP). Neighborhoods were calculated using the pp.neighbors() Scanpy function and the UMAP conectivities were computed with the Scanpy tl.umap() function as euclidean distances. From the obtained projection, Leiden clustering was performed with the Scanpy function tl.leiden() with a resolution of 1. Integration of different samples was performed on normalized PCA data using scVI tools (v.0.19.0) previous to Leiden clustering. Integrated clusters were classified as different cell types based on the expression level of specific marker genes per cluster. Cells identified as metaplastic belonging to the 15 months PTI sample were automatically categorized as tumoral cells. Python packages Numpy, Pandas and Matplotlib (7) were used for data formatting and processing. Both Scanpy and Seaborn (8) were both used for data visualization.

RNA expression data of human normal/PDAC epithelial cells (GSE134217) were obtained from fresh primary untreated PDAC specimens and distal adjacent normal pancreas by cell sorting. Epithelial cells were defined as EPCAM^{+} (CD326-FITC, 1:11, Clone: AC128, Miltenyi Biotec), and CD45^{-} (CD45-VioBlue, 1:11, Clone: 5B1, Miltenyi Biotec) after cell death exclusion using propidium iodide. Patients received partial pancreatoduodenectomy at the Department of General, Visceral and Transplantation Surgery, University of Heidelberg. Patients were part of the HIPO-project. The study was approved by the ethical committee of the University of Heidelberg (case number S-206/2011 and EPZ-Biobank Ethic Vote #301/2001) and conducted in accordance with the Helsinki Declaration; written informed consent was obtained from all patients. RNA extraction, library preparation and RNA sequencing were performed as previously described (9).

Single cell RNA-seq from treatment-naïve human PDAC samples were obtained from the Hwang *et al.* study (GSE202051 and GSE199102) (10). Data was downloaded through the Single cell Data Portal from Broad Institute (https://singlecell.broadinstitute.org/single_cell) with cell annotations. R package Seurat v5.0.1 was used for analysis of the data. Tumor cells were filtered, and cells were annotated as PGLYRP1+ when gene expression was higher than 0. FindAllMarkers function was used to generate the markers list for PGLYRP1^{+} and PGLYRP1^{-} tumor cells. The final list of PGLYRP1+ cells markers was performed by filtering genes with log2FC>1 and FDR-pval<0.05. Visualization of gene expression through the different cell-types and clusters were as

performed using UMAP, ViolinPlot and DotPlot functions.

RNA isolation for microarray analysis. For the analysis of mouse tumor-derived cell populations, Lineage$^-$ (CD45$^-$CD31$^-$), EpCAM$^+$Sca-1$^-$CD133$^-$, EpCAM$^+$Sca-1$^-$CD133$^+$, EpCAM$^+$Sca-1$^+$CD133$^-$ and EpCAM$^+$Sca-1$^+$CD133$^+$ tumor cells (~10,000 per array) were directly FACS-sorted into Trizol Reagent (Invitrogen) and RNA was purified using manufacturer's instructions with the exception of the addition of 3 µg of linear polyacrylamide (Applied Biosystems) preceding the precipitation step. Three biological replicates were obtained for each tumor population. For the comparison of mouse primary tumor cell lines grown as adherent monolayers or as spheroids (conditions detailed below), 5,000 cells/ml (10 ml) were grown for 72 h in tissue-culture dishes or in ultra-low attachment dishes (Corning). After 72 h, cells were harvested in Trizol Reagent (Invitrogen) and RNA was purified using the manufacturer's instructions. Isolated RNA was further purified using the RNeasy MinElute Cleanup kit (Qiagen) following the manufacturer's instructions.

General RNA extraction and RT-qPCR analysis. RNA was extracted using guanidine thiocyanate as described before (11) and cDNA synthesized with the SuperScript IV VILO cDNA synthesis kit (Cat. no 11756050, Invitrogen) followed by RT-qPCR analysis using SYBR green and a StepOne Plus real-time thermo-cycler (Applied Biosystems). The thermal cycle conditions consisted of an initial denaturation step of 10 min at 95°C followed by 40 cycles of denaturation (15 s at 95°C) and annealing (1 min at 60°C). The results obtained for each gene were normalized with the β-actin or HRPT levels. Primers are listed in **Supplementary Table 2**.

Western Blot analysis. Cells were harvested and lysed in RIPA buffer (Sigma, Cat no. R0278-50ML) supplemented with a protease inhibitor cocktail (Roche Applied Science, Indianapolis, IN). Cell supernatants were concentrated using the Millipore Centriplus centrifugal concentrator 30kDA (Millipore, Cat. no. 4423), as per the manufacturer's instructions. Fifty µg of protein were resolved by SDS-PAGE and transferred to Amersham™ HybondR P Western blotting PVDF membranes (Sigma, Cat no. 10600021). Membranes were sequentially blocked with 1X PBS containing 5% BSA (w/v), incubated with a 1:500-1:5000 dilution of indicated antibodies (see **Supplementary Table 3**) overnight at 4°C, washed 3 times with 1X PBS containing 0.5% Tween20 (v/v), incubated with horseradish peroxidase-conjugated secondary antibodies, and washed again to remove unbound antibody. Bound antibody complexes were detected with SuperSignal chemiluminescent

substrate (Pierce, Cat no. 34580) and images were obtained using the MyECL or iBright Imager (Thermo Fisher Scientific). Densitometric ratios were determined using ImageJ software and by measuring the intensity of each band and normalizing to total protein (i.e., No Stain Protein Labeling Reagent, Themo Fisher Scientific, Cat no. A44717; Ponceau S solution, Sigma, Cat no. P7170) or a specific housekeeping loading control.

Spheroids culture. Spheroids enriched in CSCs were generated by culturing cancer cells in ultra-low attachment plates (Corning) using serum-free DMEM/F12 (Invitrogen, Cat no. 21331046) supplemented with B27 1:50 (Invitrogen, Cat no. 17504044), 20 ng/ml bFGF (PAN-Biotech, Sigma, Cat no. GF446-10UG), L-Glutamine (Invitrogen, Cat no. 25030081), 50 units/ml penicillin/streptomycin (Invitrogen, Cat no. 11548876) and fungizone (Invitrogen, Cat no. 15290018). For spheroid formation assays, pancreatic CSC spheroids were generated by culturing primary pancreatic cancer cells (2,000 cells/ml) using the aforementioned medium. For serial passaging, 4-7-day-old spheroids were harvested using 40 µm cell strainers, dissociated into single cells with trypsin, and then re-cultured for 4-7 additional days. Spheroid formation was quantified using a Casy TTC cell counter (OMNI Life Science GmbH & Co KG, Bremen, Germany). Counts > 40µm were considered spheroids. For infliximab (Cat no. Y0002047, Sigma-Aldrich) treatment, spheroids were cultured for 4-7 days and then treated with 10 µg/ml for 24 hours. After that, they were processed for further analysis as indicated.

Organoid formation. $2x10^4$ sorted cells were plated per well in a 24-well plate in semi-solid growth factor reduced Matrigel™ (Cat no. 356234, Corning) with culture medium (DMEM-F12 supplemented with B-27, 10ng/ml EGF (PeproTech, Cat no. 315-09), 5% fetal calf serum, 50 units/ml penicillin/streptomycin). After 1 day, medium was changed to serum-free medium, and cells were incubated for 10 more days.

Single cell suspension preparation. Tumors and pancreata were digested with Collagenase P (Roche, Cat no. 11213857001), to obtain single cell suspensions. Briefly, tissues were minced using a scalpel and forceps and transferred to a 50ml conical tube. Five ml of FBS-free medium and 50 µl of Collagenase P 1X (0.2mg/ml), Dispase (0.8 mg/ml) and DNAse (0.5 mg/ml) were added to the tubes. Samples were incubated for 15 min at 37°C, vortexing every 5 min. After incubation, digestions were stopped by adding 1 ml of HBSS containing 10% FBS. Tubes were centrifuged for 5 min at 1500 rpm and supernatants were removed. ACK lysis buffer was used to remove erythrocytes by incubating samples for 5 min at room temperature, HBSS was then added, and

samples were centrifuged, supernatant removed, and cell pellets resuspended in flow cytometry buffer (PBS with 2%FBS and 3 mM EDTA).

Flow cytometry and cell sorting. For analysis of murine cells, cell suspensions were incubated with purified anti-mouse CD16/CD32 (Fc Shield,2.4G2, Cat no. 70-0161-U100, TONBO) to block Fc receptors, while for the analysis of human cells, cell suspensions were blocked with Flebogamma (Grifols) for 15min at 4°C. For cell surface marker expression, refer to antibodies listed in **Supplementary Table 4**. For all assays, 2µg/ml DAPI (4',6-diamidino-2-phenylindole, Sigma, Cat no. D9542-5MG), 1 µg/ml PI (3,8-Diamino-5-[3-(diethylmethylammonio)propyl]-6-phenylphenanthridinium diiodide Phenanthridinium, Alfa Aestar, Cat no. J66584), 1µl/mL LIVE/DEAD™ Fixable Green Dead Cell Stain (Thermo Fisher Scientific, Cat no. L23101) or 1 µg/ml TO-PRO-3 iodide (Thermo Fisher Scientific, Cat no. T3605) were used to exclude dead cells. Cells were resuspended in Flow buffer [1X PBS; 3% FBS (v/v); 3mM EDTA (v/v)] before analysis. All samples were analyzed using a 4-laser Attune™ NxT Acoustic Cytometer (Thermo Fisher Scientific) or sorted using a FACS MoFlo Astrios instrument (Becton Dickinson). Data analysis was performed using FlowJo software (Tree Star Inc., Ashland, OR.).

Histopathology and tissue microarrays. Specimens were fixed in 10% buffered formalin and embedded in paraffin or OCT. For histopathological analyses, tissues were serially sectioned, and every 4th section was stained with hematoxylin and eosin (H&E). Representative sections were chosen for the grading and enumeration of lesions and quantification of tissue damage. For H&E-stained slides of pancreata, areas were marked as normal tissue, ADM, PanIN or PDAC tumor tissue. Moreover, tumors were classified as differentiated or poorly differentiated using the guidelines from the College of American Pathologists (https://documents.cap.org/protocols/cp-pancreas-exocrine-17protocol-4001.pdf). For macroscopic images of tumors or metastatic tissue, a Motic EasyScan One was used to acquire whole images and images were scaled and annotated with Motic DS Assistant software. For the TMAs, human PDAC tumor tissue samples were provided by the tissue bank of the University Medical Center Mainz in accordance with the regulations of the tissue biobank and the approval of the ethics committee of University Medical Center Mainz (2019-14390; Landesärztekammer RLP). Tissue samples of 113 patients with primary resected therapy naive PDAC

were spotted on tissue microarrays (TMA). To overcome heterogeneity, 4 array spots of each tissue were generated (2 central, 2 peripherals; diameter: 1 mm). Prior to incubation with the primary antibody, heat-induced antigen retrieval was conducted using citrate-based buffer (pH 6.0 Dako EnVision, Glostrup, Denmark). Then, a primary mouse moncolonal anti-hPGLYRP1 antibody (Cat no. MAB2590, R&D) was used at a dilution of 1:250. Antibody-binding was visualized with DAB+ chromogen (Dako). The stained tissue slides were scanned at 400× magnification using a slide scanner (NanoZoomer 2.0-HT, Hamamatsu Photonics K.K., Hamamatsu, Japan). Analysis was performed using NDP2.view software (Hamamatsu). The staining was quantified by using semi-quantitative and the widely established Allred scoring system, the sum of staining intensity and distribution. Tissue samples were provided by the tissue bank of the University Medical Center Mainz in accordance with the regulations of the tissue biobank and the approval of the ethics committee of University Medical Center Mainz.

Immunofluorescence and confocal microscopy. Spheroids were stained as described by the M.J. Bissell laboratory (12). Briefly, cells were spun down and washed once before resuspension in 15µl of PBS, plated on super-frost slides, dried, and fixed in 4% formaldehyde. After permeabilization with 0.3% Triton X-100 in PBS, slides were incubated in blocking solution (PBS, 0,1% BSA, 10% horse serum, 1% donkey F(ab') anti-mouse IgG (Cat no. 715-006-151, Jackson Immunoresearch), 0.2% Triton X-100, 0.05% Tween 20) and then stained.

For staining of pancreata and tumors embedded in OCT, samples were sectioned (6µm) and fixed in 4% PFA. When required, sections were permeabilized with 0.3% Triton X-100 for 10 minutes and washed with PBS. After incubation with blocking solution (3% BSA, 1% donkey serum, 0.05% Tween 20, 0.1% Triton X- 100), primary antibodies (**Supplementary Table 3**) were added and incubated overnight at 4°C followed by a 1h incubation with secondary antibodies and DAPI. Images were obtained using a TCS SP5 confocal microscope (Leica). For 2D sections in paraffin, FFPE blocks were cut using a Leica RM2125 RTS microtome, sections were deparaffinated with decreasing concentrations of ethanol and antigen retrieval with 1 mM EDTA was used. After incubation with blocking solution (PBS 1x, 1% BSA, 2% FBS), primary antibodies were added and incubated overnight at 4°C, followed by a 1h at 37°C incubation with secondary antibodies and DAPI. Samples were mounted in ProLong (Invitrogen, Cat no. P36970) and images were obtained using a Confocal Laser

Scanning Microscope LSM710 (Zeiss) or Stellaris 8 STED microscope (Leica). Images were analyzed in ImageJ software. For 3D quantitative confocal microscopy, FFPE-embedded tumors were deparaffinized as described before (20 µm slides) or following already published protocols (13) and cut using a Leica Vibratome VT1200 (300 µm slides). Tissue sections were blocked, incubated, and cleared as previously described (14), and images were obtained using a Stellaris 5 confocal microscope (Leica). Surfaces and spots were created and quantified employing Bitplane Imaris ® v10, and distances between spots and surfaces were measured.

Trans-well migration assay. Invasion assays were performed using 8.0 µm transparent PET membrane trans-well chambers (Cat no. 353097, Falcon). $1x10^5$ murine immortalized bone marrow-derived macrophages (kindly provided by Dr. Antonio Castrillo), freshly isolated neutrophils or NK-92 cells were added to the top of the insert, and 750 µl of complete RPMI media (as control media) or media plus recombinant PGLYRP1 (1µg/ml, BioTechne, Cat no. 2696-PG-050/CF) was added to the lower chamber. The assay chambers were incubated for 6-24h at 37°C. Invaded macrophages were fixed in 4% PFA and stained with Crystal Violet, and the number of cells that migrated through to the lower chamber were counted. For neutrophils, migrated and non-migrated cells were collected and stained with Anti-Ly6G antibody and analyzed by flow cytometry as described above.

Lentivirus production and transduction. Lentiviral particles were produced by transfection of 293T cells (Invitrogen) following a polyethylenimine (PEI)-based protocol, as previously described (15). Briefly, 8.5×10^5 293T cells were co-transfected with 1 µg packaging plasmid psPAX2, 1 µg envelope plasmid pVSVG and 2 µg of the indicated backbone plasmid: pRRL-CMV-IRES-mCherry, pReceiver-Lv106_PGLYRP1 (murine PGLYRP1, 217EX-Mm05789-Lv206, GeneCopoeia), pReceiver-Lv106_PGLYRP1 (human PGLYRP1, 217EX-U0257-Lv206, GeneCopoeia), pCAS9 or 5 different PGLYRP1-CRISPR plasmids (Target sequences: For murine PGLYRP1 were CATGACCGTCCTCTCCAATA, AGGCGGCTAGAGCACTCGGA and ATGTCTATGAAGGCCGAGGC; for human PGLYRP1 were GATACCACCACATAGCGTAA and GAAGACGGGCTCGTATACGA; and they were cloned into pKLV2-U gRNA5(BbsI)-PGKpuro2ABFP-W backbone vector from Addgene (#67974)) or control scrambled sgRNA CRISPR Lenti-vector (Addgene, #67974). After 8 h, the transfection medium was replaced with complete RPMI media and recombinant lentiviruses were harvested 48 h and 72 h later. Virus particle-containing

supernatants were filtered through 0.45 µM PVDF membrane filters, aliquoted and stored at −80 °C until needed. For lentivirus transduction, PDAC cells were seeded in 6-well plates at a concentration of 3–5 × 10^5 cells/well. One milliliter of the respective lentivirus was directly overlaid onto cells in the presence of polybrene (Cat. no TR-1003, Sigma) at a final concentration of 8 µg/ml. After 16 h, medium was changed and after 48 h, antibiotic selection was initiated. Stably-transduced cells were obtained after BFP- or mCherry-positive cell sorting using a FACS Vantage SE Flow Cytometer for PGLYRP1-CRISPR, controls and PGLYRP1 overexpressing cells, respectively, or after antibiotic cell selection using Blasticidin (5–10 µg/ml; Cat. no R21001, Invitrogen) (Cas9) or Puromycin (2 µg/ml; Cat. no P9620, Sigma) (PGLYRP1 overexpression and CRISPR). Before their use, OE and KO cultures were antibiotic-selected to ensure, up to a minimum, of 80% mCherry+ or BFP+ cells.

Immune cell isolation. Murine bone-marrow-derived cells (BMDC) were isolated from the femur and tibiae of at least two mice by centrifugation. BMDC pellets were resuspended in RPMI 1640 media (Invitrogen, Cat no. 61870044) and cultured in the absence of serum for 24h to allow monocytes to attach. Monocytes were then differentiated into macrophages under adherent conditions on non-tissue culture-treated 100mm dishes in RPMI supplemented with 10% FBS and 10ng/ml of murine M-CSF (Cat no. 315-02, PeproTech). Murine neutrophils were isolated from spleens or tumors and sorted based on the expression of the markers Ly6C and Ly6G.

Blood samples from healthy donors were provided by the BioBank Hospital Ramón y Cajal-IRYCIS (PT13/0010/0002), integrated in the Spanish National Biobanks Network (ISCIII Biobank Register No. B.0000678). Samples were processed following standard operating procedures with the appropriate approval of the Hospital Ramón y Cajal Ethical and Scientific Committee (Control No.: DE-BIOB-73 AC65, RG.BIOB-57, and RG.BIOB-54), with informed consent and according to Declaration of Helsinki principles. Blood samples were diluted with PBS (Cat no. 10010023, Gibco) and Ficoll (Cat no. L6115, Merck) and were used to isolate peripheral blood mononuclear cells (PBMCs). PBMCs were divided across three 6-well culture plates (per donor) with RPMI 1640 media containing 10% FBS and 50 units/ml penicillin/streptomycin. After

24 h, monocytes adhered to the plate surface were separated from the lymphocytes that remained in suspension and that were seeded in T-75 flasks for their subsequent activation and growth.

Macrophage phagocytosis assay. Five days after isolation of macrophages, tumor fluorescent cells were harvested and seeded together with macrophages during 24 h. In case of Infliximab and anti-PGLYRP1 treatment, cells were incubated with 10 µg/ml or 0.5 µg/ml, respectively. Afterwards, cells were detached using scrapers and macrophages phagocytosing fluorescent tumor cells were measured by flow cytometry, as described above, gating on the double-positive population: CD11b and reporter genes (i.e., mCherry or BFP).

T cell cytotoxicity assay. Murine T cells were isolated from spleens and cultured overnight at 37°C in a humidified incubator with 5% CO_2 with mouse T-Activator CD3/CD28 Dynabeads (ThermoFisher Scientific) and recombinant mouse IL-2 (30 U/ml, Cat. no 212-12, Peprotech) for at least 48 hours to achieve a T cell enrichment of at least 80%. Seven days after isolation and expansion of human T cells from PBMCs (described above), T cells were activated with phytohemagglutinin-P (Cat no. L8754, Merck) at a concentration of 5 µg/ml and with 20 ng/ml of human rIL-2 (Cat no. PHC0026, Gibco) for 7 days. Subsequently, fluorescent WT, OE or CRISPR human or murine tumor cells were incubated with T cells (or medium control) in wells of a 96-well plate in a 1:5 ratio for 24 h at 37°C in a humidified incubator with 5% CO_2. Afterwards, cytotoxicity was determined by quantifying dead tumor cells (labeled with mCherry or BFP), using TO-PRO-3 staining by flow cytometry and a comparison between the excess of cell death compared to the controls (i.e., background) was performed.

Immune cell polarization assays. For macrophage polarization, primary bone marrow-derived MΦs were cultured with 10 ng/ml of IL-4 (Cat no. 214-14, PeproTech) for 48h (M2), LPS (Cat. no L4391, Sigma) + IFNγ (Cat. no 315-05, Peprotech) (M1) or recombinant PGLYRP1 (1 µg/mL). Macrophage polarization was confirmed by qPCR analysis of indicated mRNAs. For neutrophil polarization, Ly6G$^+$ neutrophils were isolated from spleens by FACS and cultured with RPMI media or RPMI media plus recombinant PGLYRP1 (1 µg/mL) or KPC cell lines conditioned media. CXCR2 or CD95 expression was measured by flow cytometry. For T cell viability and polarization, T cells were enriched from a splenocyte culture and sorted for CD3 expression. 100,000 T cells were cultured with RPMI or RPMI media plus recombinant PGLYRP1 (1 µg/mL) with or without previous

T cell activation as described above. Cytotoxicity was measured using DAPI and polarization was measured by flow cytometry.

Stimulation assay. For stimulation assays, tumor or immune cells were seeded in 6-well plates and treated with 1 ml of complete RPMI medium plus recombinant PGLYRP1 (1 µg/mL, Cat. no. 2696-PG-050/CF, Biotechne) and/or TNFα (20 ng/mL, Cat. no. MBS2553644) during 6 or 24h, or with macrophage conditioned medium for 24h. After indicated time points, cells were trypsinized and harvested for flow cytometry analysis of cytotoxicity "excess" (i.e., the difference between basal cell death levels and those after stimulation) and/or expression of the indicated markers.

Mice. The KPC (LSL-Kras$^{G12D/+}$;LSL-Trp53$^{R172H/+}$;Pdx-1-Cre) mouse model of PDAC has been previously described (16). All *in vivo* procedure in mice were conducted in accordance with protocols approved by the local Animal Experimental Ethics Committee of the Instituto de Salud Carlos III (PA 34-2012) or the Committee for Animal Care from the Universidad Autónoma de Madrid (UAM) (Ref# CEI-25-587) and the Comunidad de Madrid (PROEX 335/14, 294/19, 289/14 or 201.4/23). For all *in vivo* experiments, mice were housed according to institutional guidelines and all experimental procedures were performed in compliance with the institutional guidelines for the welfare of experimental animals and in accordance with the guidelines for Ethical Conduct in the Care and Use of Animals as stated in The International Guiding Principles for Biomedical Research involving Animals, developed by the Council for International Organizations of Medical Sciences (CIOMS).

Extreme Limiting Dilution Analysis. For assessing tumorigenic potential of the different tumor cell subsets, serial dilutions of sorted cells were injected into the flanks of athymic Foxn1nu/nu mice (Envigo). Cells were embedded in a mixture 1:1 Medium:Matrigel™ in a final volume of 30 µl. Animals were examined twice a week for tumor formation for up to 2 months, and were sacrificed when the diameter of the tumor reached 1cm^2. CSC frequency was calculated using the ELDA software (17). C57Bl/6J mice were purchased from our in-house breeding facility (Instituto de Investigaciones Biomedicas Sols-Morreale CSIC-UAM). Three dilutions (100,000, 10,000 and 1,000 cells) of KPC ID11 and ID95 WT, PGLYRP1 OE and PGLYRP1 KO cell lines were subcutaneously injected into 10-week-old female C57Bl/6J mice and tracked for 3 weeks for tumor formation. At the time of sacrifice, tumors were extracted, photographed, weighed, and fixed in 4% PFA overnight at 4°C and

subsequently paraffin embedded.

Murine model of pancreatitis. Pancreatitis was induced by treatment with Cerulein (Cat no. C9026, Sigma), a cholecystokinin analog, as a single daily intraperitoneal injection (0.1 ml of a 50 µg/ml solution in saline buffer) 5 days per week during the duration of the study in C57Bl6 mice. After treatment, mice were euthanized, and inflammation was checked by the presence of acinar to ductal metaplasia in H&E-stained slides from FFPE-embedded pancreata.

Intravenous metastasis assay. For lung colonization assays, C57Bl/6J mice were injected intravenously via the tail vein with 10^6 KPC PGLYRP1 WT, OE or KO cells (resuspended in 100 µl 0.9% physiological saline solution) using a 27-G needle. Lungs were harvested 3 weeks post injection, photographed, weighed, fixed in 4% PFA overnight at 4 °C and subsequently paraffin embedded.

Zebrafish maintenance and xenograft assays. Zebrafish adults (*Danio rerio*, wild type) were crossed to obtain embryos. Adults were kept in aquaria with a ratio of 1 fish / L of water, day/night cycle of 14:10h, respectively; and water temperature was maintained at 28.5°C, according to published procedures (18). Procedures used in the experiments, fish care and treatment were performed in agreement with the Animal Care and Use Committee of the University of Santiago de Compostela and the standard protocols of Spain (Directive 2012-63-DaUE). At the end of the experiments, the embryos used were euthanized by tricaine overdose.

Zebrafish embryos were obtained mating adults and collected at 0hpf (hours post fertilization) and placed in an incubator at 28.5°C in petri dishes until 48hpf. On the day of the experiment, tumor cells were trypsinized, labeled with DiI (Cat. no D3911, Thermo Fisher) and concentrated in an Eppendorf at approximately 1 million cells/condition and resuspended in 10µL of PBS with 2% of PVP40) to avoid cellular aggregation. Afterwards, 2-day-old embryos were anesthetized with 0.003% of tricaine (Cat. no E10521, Sigma) and injected with PANC354 or PANC185 cells (WT or CRISPR). Cell injection was performed using borosilicate needles (1 mm O.D. x 0.75 mm I.D.; World Precision Instruments). Between 100 and 200 cells were injected into the circulation of each fish (Duct of Cuvier) using a microinjector (IM-31 Electric Microinjector, Narishige) with an output pressure of 15kPA and 20ms of injection time per injection. After the injection, embryos were maintained at 34°C until 6 days post injection (dpi) in 30mL petri dishes with SDTW (Salt Dechlorinate Tap

Water).

Imaging of the injected embryos was performed using a fluorescence stereomicroscope (AZ-100, Nikon) at 1, 4 and 6dpi to measure the proliferation and metastasis capacity of the injected cells in the caudal hematopoietic tissue (CHT) of the zebrafish embryos in each of the conditions assayed. In order to perform the analysis of the images of the embryos taken at the different time points (1, 4 and 6 dpi), Quantifish software (19) was used. This software processes all the images and measures fluorescence intensity and area of positive pixels, corresponding to the cells injected, above a certain threshold. With these parameters, integrated density is obtained allowing one to compare different times between images and obtain a proliferation ratio of the cells in the region of the caudal hematopoietic tissue (CHT) of the embryos, where the cells normally metastasize. A ratio equal to 1 means the maintenance of the cells, above 1, the cells are proliferating and below 1 the cells are dying. In addition, the number of tumors present in each image corresponding to the tails of the embryos were counted. Tumor counts were selected based on the size of the tumor and the separation between the cell masses in the tail of the fish.

PGLYRP1 Enzyme-Linked ImmunoSorbent Assay. Blood samples from PDAC patients and healthy donors were provided by the BioBank Hospital Ramón y Cajal-IRYCIS (PT17/0015/0010), integrated in the Spanish National Biobanks Network (ISCIII Biobank Register No. B.0000678) and from the "Collection of samples of the Familial Pancreas Cancer Registry" of the Carlos III Institute (ISCIII ref no.: C.0003953). PGLYRP1 levels in serum samples were determined using the human PGLYRP1 ELISA kit (Cat no. EHPGLYRP1, Invitrogen) as per the manufacturer's instructions.

TNFα Enzyme-Linked ImmunoSorbent Assay. Conditioned medium from KPC cells grown in adherent conditions or as spheroids were collected, and the levels of soluble TNFα were determined using the murine TNFα ELISA kit (Cat no. DY410-05, R&D) as per manufacturer's instructions.

Patient and public involvement in research. Neither patients nor the public were directly involved in this research. Included are patient samples that were provided with informed consent via the BioBank Hospital Ramón y Cajal-IRYCIS (detailed above).

BMJ Publishing Group Limited (BMJ) disclaims all liability and responsibility arising from any reliance placed on this supplemental material which has been supplied by the author(s)

The Peptidoglycan Recognition Protein 1 Confers Immune Evasive Properties on Pancreatic Cancer Stem Cells

Supplementary Tables

Supplementary Table 1. Custom gene signatures employed

Name	Number of genes	Reference
BENPORATH_ES1	379	PMID: 18443585
ZHOU_PANCREATIC_EXOCRINE_PROGENITOR	11	PMID: 18754011
RAMALHO_STEMNESS_UP	196	PMID: 12228720
WONG_ADULT_TISSUE_STEM_MODULE	721	PMID: 18397753
WONG_EMBRYONIC_STEM_CELL_CORE	335	PMID: 18397753
STEM CELL GENE SET (Palmer et al. 2012)	189	PMID: 22909066
IVANOVA_HEMATOPOIESIS_STEM_CELL	191	PMID: 12228721
HEMATOPOIETIC STEM CELL HOMEOSTASIS	33	GO:0061484
BOQUEST_STEM_CELL_UP	262	PMID: 15635089
KRASP53 PDAC IMMUNEREGULATORY PROGRAM (DATTA ET AL)	20	PMID: 35701533
REACTOME_INNATE_IMMUNE_SYSTEM	278	R-HSA-168249
LIN_TUMOR_ESCAPE_FROM_IMMUNE_ATTACK	19	PMID: 17308126
INVASIVE_GENE_SIGNATURE_LIU_ET_AL_NEJM	186	PMID: 17229949

Supplementary Table 2. Primers

Gene	Species	Sequence Forward	Sequence Reverse
Arginase1	Mouse	TCATTTGGGTGGATGCTCACAC	GAGAATCCTGGTACATCTGGGAA
B-ACTIN	Human	GCGAGCACACGAGCCTCGCCTT	CATCATCCATGGTGAGCTGGCGG
B-Actin	Mouse	CGGTTCCGATGCCCTGAGCTCTT	CGTCACACTTCATGATGGAATTGA
Camp	Mouse	GCAGTTCCAGAGGGACGTC	GTTCCTTGAAGGCACATTGC
Ccl2	Mouse	CACTCACCTGCTGCTACTCA	GCTTGGTGACAAAAACTACAGC
Ccl7	Mouse	GATCTCTGCCACGCTTCTGT	GGTGATCCTTCTGTAGCTCTTG
Cd86	Mouse	ACGTATTGGAAGGAGATTACAGCT	TCTGTCAGCGTTACTATCCCGC
Csf1	Mouse	GCCTCCTGTTCTACAAGTGGAAG	ACTGGCAGTTCCACCTGTCTGT
Il1b	Mouse	TGGACCTTCCAGGATGAGGACA	GTTCATCTCGGAGCCTGTAGTG
Krt19	Mouse	AGTCTCGCTGGTAGCTCAGATG	CTACCTTGCTCGGATTGAGGAG
Pdx1	Mouse	AAGCTCACGCGTGGAAAG	GCCGGGAGATGTATTTGTTAAA
PGLYRP1	Human	CCATGTCCATTGGCATCAGCTTC	GAGCACATAGTTGGACCTCAGG
Pglyrp1	Mouse	CAATGTGCAGCATTACCACA	CCGTCCTCTCCATAAGGAA
Sox9	Mouse	TCCACGAAGGGTCTCTTCTC	AGGAAGCTGGCAGACCAGTA
Tnf	Mouse	TCGGGGTGATCGGTCCCCAA	TGGTTTGCTACGACGTGGGCT
Ym1	Mouse	GGGCATACCTTTATCCTGAG	CCACTGAAGTCATCCATGTC
Trem1	Mouse	CCTGTTGTGCTCTTCCATCCTG	CGGGTTGTAGTTGTGTCACTGG
Mpo	Mouse	CGTGTCAAGTGGCTGTGCCTAT	AACCAGCGTACAAAGGCACGGT
Tnfrsf1a	Mouse	GTGTGGCTGTAAGGAGAACCAG	CACACGGTGTTCTGAGTCTCCT
Cxcr2	Mouse	CTCTATTCTGCCAGATGCTGTCC	ACAAGGCTCAGCAGAGTCACCA
Cxcr4	Mouse	GACTGGCATAGTCGGCAATGGA	CAAAGAGGAGGTCAGCCACTGA

Supplementary Table 3. Western Blot and immunofluorescence antibodies

Western Blot/Immunofluorescence					
**Epitope**	**Host**	**Reactivity**	**Dilution**	**Application**	**Manufacturer (Cat no).**
**Amylase**	Rabbit	Mouse	1:100	IF	Sigma (A8273)
**CD133/1**	Mouse	Human	1:100	IF	Miltenyi (130-111-756)
**CD133**	Rabbit	Mouse	1:200	IF	Abcam (ab19898)
**CD133-APC**	Rat	Mouse	1:100	IF	eBioscience (17-1331-81)
**CD3**	Rat	Mouse	1:100	IF	eBioscience (14-0032-82)
**CD4**	Rat	Mouse	1:100	IF	eBioscience (14-0041-82)
**CD45-FITC**	Rat	Mouse	1:50	IF	BD (553080)
**EpCAM**	Rabbit	Mouse, Human	1:100	IF	Abcam (ab71916)
**EpCAM**	Rat	Mouse	1:100	IF	Biolegend (118202)
**F4/80**	Rabbit	Mouse	1:100	IF	Proteintech (28463-1-AP)
**Hes1**	Mouse	Mouse	1:100	IF	SantaCruz (sc-166410)
**Insulin**	Mouse	Mouse	1:100	IF	SantaCruz (sc-8033)
**Krt19**	Mouse	Mouse	1:100	IF	Abcam (ab220193)
**MPO**	Rabbit	Mouse	1:100	IF	Abcam (ab9535)
**NK1.1-PE**	Mouse	Mouse	1:50	IF	BioLegend (156504)
**PanCK**	Mouse	Mouse, Human	1:200	IF	Sigma (C5992)
**Sca-1-PE**	Rat	Mouse	1:100	IF	eBioscience (12-5981-82)
**Secondary Alexa Fluor 488**	Donkey	Rat	1:200	IF	Invitrogen (A21208)
**Secondary Alexa Fluor 546**	Donkey	Rabbit	1:200	IF	Invitrogen (A31572)
**Secondary Alexa Fluor 555**	Donkey	Rabbit	1:200	IF	Invitrogen (A31572)
**Secondary Alexa Fluor 568**	Donkey	Mouse	1:200	IF	Invitrogen (A10040)
**Secondary Alexa Fluor 647**	Donkey	Goat	1:200	IF	Invitrogen (A21447)
**Secondary Alexa Fluor 647**	Donkey	Rabbit	1:200	IF	Invitrogen (A32795)
**Secondary Alexa Fluor 647**	Donkey	Mouse	1:200	IF	Invitrogen (A31571)
**Secondary Alexa Fluor 680**	Donkey	Mouse	1:200	IF	Invitrogen (A10038)
**Secondary AlexaFluor 488**	Donkey	Goat	1:200	IF	Invitrogen (A11055)
**Secondary AlexaFluor 594**	Donkey	Rat	1:200	IF	Thermo Fisher (A21209)
**Streptavidin-AF488**			1:800	IF	Molecular Probes, S11223
**GAPDH**	Mouse	Mouse, Human	1:5000	WB	Thermo Fisher (AM4300)
**Secondary HRP**	Donkey	Goat	1:5000	WB	Rockland (605-703-002)
**Secondary HRP**	Sheep	Mouse	1:5000	WB	Amersham (NA931)
**Secondary HRP**	Donkey	Rabbit	1:5000	WB	Amersham (NA934)
**Tubulin**	Mouse	Mouse, Human	1:5000	WB	Elabscience (E-AB-20033)
**PGLYRP1**	Goat	Mouse	1:100-1:1000	WB/IF	R&D (AF2696)
**PGLYRP1**	Goat	Human	1:1000	WB/IF	R&D (AF2590)

Supplementary Table 4. Flow cytometry antibodies

Host	Reactivity	Target	Fluorochrome	Company	Cat. No.	Dilution
**rat**	human/mouse	CD11b	PerCpCy5.5	TONBO bioscience	65-0112	0.25/50
**rat**	human/mouse	CD133	FITC	eBioscience	11-1331-82	5/50
**mouse**	human	CD133/1	Vio Bright R667	Miltenyi	130-111-756	2.5/50
**rat**	mouse	CD133/1	APC	Miltenyi	130-102-197	5/50
**rat**	mouse	CD206	PE	Thermo Scientific	12-2061-80	0.5/100
**rat**	mouse	CD31	FITC	BD	553372	1/50
**mouse**	mouse	CD31	Pacific Blue	ThermoFisher	RM5228	1/50
**hamster**	mouse	CD3ε	APC-Cy7	BioLegend	100330	1.25/50
**rat**	mouse	CD4	PE	BioLegend	100512	0.5/50
**rat**	mouse	CD45	PE-Cy7	eBioscience	25-0451-82	0.6/100
**mouse**	mouse	CD45	Pacific Blue	ThermoFisher	MCD4528	0.6/100
**rat**	mouse	CD8	APC	BioLegend	100712	1/100
**mouse**	human	CD86	PerCP-Cy5.5	BioLegend	374215	1/100
**mouse**	mouse	CD86	APC	Miltenyi	130-102-558	5/50
**mouse**	mouse	CD95	PE	eBioscience	12-0951-81	2.5/50
**rat**	mouse	CXCR2	APC	Biolegend	149311	2.5/100
**mouse**	human	CXCR4 (CD184)	PE	Miltenyi	130-117-354	1/50
**rat**	mouse	EpCAM (CD326)	PE	Miltenyi	130-102-265	1/50
**mouse**	human/mouse	EpCAM (CD326)	APC	Biolegend	324208	1/50
**rat**	mouse	F4/80	APC	Miltenyi	130-102-379	1/100
**hamster**		IgG	APC	BioLegend	400911	
**hamster**		IgG	APC-Cy7	Biolegend	400927	
**human**		IgG1	APC	Miltenyi	130-113-446	
**rat**		IgG1 K	AF700	Biolegend	400420	
**rat**		IgG1 K	PE	BioLegend	400407	
**mouse**		IgG2a K	PE	BioLegend	400211	
**rat**		IgG2a K	PE	BioLegend	400507	
**rat**		IgG2a K	AF700	Invitrogen	56-4321-80	
**Rat**		IgG2a K	APC	BioLegend	400511	
**Rat**		IgG2b K	PE	BioLegend	400607	
**Rat**		IgG2b K	PE-Cy7	Biolegend	400617	
**rat**		IgG2b K	PerCP-Cy5.5	eBioscience	45-4031-80	
**rat**		IgG2c K	APC-Cy7	Biolegend	400719	
**mouse**		IgG3 K	FITC	Biolegend	401317	
**rat**	mouse	Ly-6C	APC-Cy7	BioLegend	128025	1.25/100
**rat**	mouse	Ly-6G	AF700	BioLegend	127621	0.5/100
**hamster**	mouse, human	PD1 (CD279)	PE	eBioscience	12-9985-81	1/50
**mouse**	human	PDL1	A700	R&D	FAB1561N	5/50
**rat**	mouse	PDL1	APC	Biolegend	124311	1/100
**mouse**	human/mouse	PGLYRP1	AF488	NovusBio	NB100-56719AF488	1/50

**rat**	human/mouse	Sca-1 (Ly-6A/E)	AF700	Invitrogen	56-5981-82	1.25/50
**rat**	mouse	Sca-1 (Ly-6A/E)	PE	BD	553335	1.25/50
**human**	mouse	TIM3 (CD366)	FITC	Miltenyi	130-120-823	1/50
**human**	human	TNFR1	APC	Miltenyi	130-120-324	1/50

Supplementary Table 5. Top 100 upregulated genes in murine CSCs

Mmp7	Wfdc2	Iqgap3	Mcm4
Cldn2	Arc	Rgs3	Akr1b7
PGLYRP1	Tmem45a	Cd44	Vsig1
Cldn2	Anxa1	Cebpd	Ctgf
Sprr2a2	Myo6	Ugt1a6b	Shc2
Msln	Ly6d	Tmprss4	Rbm4b
Expi	Ecm1	Foxq1	Clca2
Ctse	Lgals4	Dclk1	Eya2
Tspan8	Kif23	Hist1h2ab	Ehf
Cxcl5	Onecut2	S100a6	Pla2g4a
Me1	Golm1	Glis3	Avil
Gcnt3	Cfi	Sprr1a	Sftpd
Ltf	Dclk1	Heg1	Cd14
Rgs17	Pld1	Inf2	Phlda1
Vcam1	Dctd	Tmeff2	S100b
Plscr1	St8sia3	Degs2	Rgs4
AA986860	Muc6	Pard3	Plekha6
Itga2	Lgals4	Eps8l3	Vcam1
Sftpd	Tinagl1	Efna5	Cxcl16
Tff1	Gpa33	Adam8	Gm4634
Mcpt2	Smpd3	Pik3cg	Ly6c1
Bace2	Pxdn	Tm4sf1	Me1
Anxa3	Tnfrsf23	P2rx2	Plac8
Slc39a4	Aadac	Vnn1	Lrrc26
Car2	Mal	Cxcr4	Mcpt8

References

1. Schlesinger Y, Yosefov-Levi O, Kolodkin-Gal D, Granit RZ, Peters L, Kalifa R, et al. Single-cell transcriptomes of pancreatic preinvasive lesions and cancer reveal acinar metaplastic cells' heterogeneity. Nat Commun. 2020;11(1):4516.
2. Nicolle R, Raffenne J, Paradis V, Couvelard A, de Reynies A, Blum Y, et al. Prognostic Biomarkers in Pancreatic Cancer: Avoiding Errata When Using the TCGA Dataset. Cancers. 2019;11(1).
3. Bailey P, Chang DK, Nones K, Johns AL, Patch AM, Gingras MC, et al. Genomic analyses identify molecular subtypes of pancreatic cancer. Nature. 2016;531(7592):47-52.
4. Martinelli P, Carrillo-de Santa Pau E, Cox T, Sainz B, Jr., Dusetti N, Greenhalf W, et al. GATA6 regulates EMT and tumour dissemination, and is a marker of response to adjuvant chemotherapy in pancreatic cancer. Gut. 2017;66(9):1665-76.
5. Zhang G, Wang Q, Yang M, Yao X, Qi X, An Y, et al. OSpaad: An online tool to perform survival analysis by integrating gene expression profiling and long-term follow-up data of 1319 pancreatic carcinoma patients. Molecular Carcinogenesis. 2020;59(3):304-10.
6. Wolf FA, Angerer P, Theis FJ. SCANPY: large-scale single-cell gene expression data analysis. Genome biology. 2018;19(1):15.
7. Hunter JD. Matplotlib: A 2D Graphics Environment. Computing in Science & Engineering. 2007;9(3):90-5.
8. Waskom ML. seaborn: statistical data visualization. The Journal of Open Source Software. 2021.
9. Espinet E, Gu Z, Imbusch CD, Giese NA, Büscher M, Safavi M, et al. Aggressive PDACs Show Hypomethylation of Repetitive Elements and the Execution of an Intrinsic IFN Program Linked to a Ductal Cell of Origin. Cancer discovery. 2021;11(3):638-59.
10. Hwang WL, Jagadeesh KA, Guo JA, Hoffman HI, Yadollahpour P, Reeves JW, et al. Single-nucleus and spatial transcriptome profiling of pancreatic cancer identifies multicellular dynamics associated with neoadjuvant treatment. Nature genetics. 2022;54(8):1178-91.
11. Chomczynski P, Sacchi N. Single-step method of RNA isolation by acid guanidinium thiocyanate-phenol-chloroform extraction. Analytical biochemistry. 1987;162(1):156-9.
12. Lee GY, Kenny PA, Lee EH, Bissell MJ. Three-dimensional culture models of normal and malignant breast epithelial cells. Nat Methods. 2007;4(4):359-65.
13. Tanaka N, Kanatani S, Tomer R, Sahlgren C, Kronqvist P, Kaczynska D, et al. Whole-tissue biopsy phenotyping of three-dimensional tumours reveals patterns of cancer heterogeneity. Nature Biomedical Engineering. 2017;1(10):796-806.
14. Gomariz A, Helbling PM, Isringhausen S, Suessbier U, Becker A, Boss A, et al. Quantitative spatial analysis of haematopoiesis-regulating stromal cells in the bone marrow microenvironment by 3D microscopy. Nat Commun. 2018;9(1):2532.
15. Sainz B, Jr., Barretto N, Martin DN, Hiraga N, Imamura M, Hussain S, et al. Identification of the Niemann-Pick C1-like 1 cholesterol absorption receptor as a new hepatitis C virus entry factor. Nature medicine. 2012;18(2):281-5.
16. Hingorani SR, Wang L, Multani AS, Combs C, Deramaudt TB, Hruban RH, et al. Trp53R172H and KrasG12D cooperate to promote chromosomal instability and widely metastatic pancreatic ductal adenocarcinoma in mice. Cancer Cell. 2005;7(5):469-83.
17. Hu Y, Smyth GK. ELDA: extreme limiting dilution analysis for comparing depleted and enriched populations in stem cell and other assays. Journal of immunological methods. 2009;347(1-2):70-8.
18. Westerfield M. The Zebrafish Book: A Guide for the Laboratory Use of Zebrafish (Danio Rerio): University of Oregon Press; 2000.
19. Stirling DR, Suleyman O, Gil E, Elks PM, Torraca V, Noursadeghi M, et al. Analysis tools to quantify dissemination of pathology in zebrafish larvae. Scientific reports. 2020;10(1):3149.
